# Cyanuric acid in *Paramecium* secretions is an efficient quorum sensing inducer

**DOI:** 10.1093/ismejo/wraf080

**Published:** 2025-08-08

**Authors:** Xingyao Ye, Xiaojun Niu, Dongqing Zhang, Mengyu Lv, Ling Li, Qiang Liu, Deye Chen, Yu Lin, Zhiquan Yang, Yi Zhang

**Affiliations:** School of Environment and Energy, South China University of Technology, Guangzhou 510006, PR China; School of Environmental Science and Engineering, Guangdong University of Petrochemical Technology, Maoming 525000, PR China; School of Environment and Energy, South China University of Technology, Guangzhou 510006, PR China; School of Environmental Science and Engineering, Guangdong University of Petrochemical Technology, Maoming 525000, PR China; The Key Lab of Pollution Control and Ecosystem Restoration in Industry Clusters, Ministry of Education, South China University of Technology, Guangzhou Higher Education Mega Centre, Guangzhou 510006, PR China; School of Environmental Science and Engineering, Guangdong University of Petrochemical Technology, Maoming 525000, PR China; School of Environment and Energy, South China University of Technology, Guangzhou 510006, PR China; School of Environment and Energy, South China University of Technology, Guangzhou 510006, PR China; School of Environment and Energy, South China University of Technology, Guangzhou 510006, PR China; China Water Resources Pearl River Planning Surveying and Designing Co., Ltd., Guangzhou 510640, PR China; Guangzhou Urban Drainage Company Limited, Guangzhou 510006, PR China; School of Environment and Energy, South China University of Technology, Guangzhou 510006, PR China; School of Environment and Energy, South China University of Technology, Guangzhou 510006, PR China

**Keywords:** quorum sensing, *Paramecium*, cyanuric acid, anammox

## Abstract

Quorum sensing is a process of bacterial chemical communication via extracellular signal molecules known as autoinducers, allowing synchronized collective behavior changes related to population density. However, the ecological significance of quorum sensing in multi-species communities, particularly coexisting with predators, remains unaddressed. In this study, we discovered that cyanuric acid (CA), a compound secreted by the widespread protozoan *Paramecium*, significantly influences the LuxR-type quorum sensing in *Pseudomonas aeruginosa* PAO1. Exogenous CA acts as a substitute for autoinducers known as N-acyl homoserine lactones in activating LasR/RhlR regulators that involve promiscuous ligand-receptor interactions, and exerts a specific restoring effect on the PAO1-Δ*lasI*/Δ*rhlI* mutant. Further, the inductive capabilities of CA were harnessed to expedite the start-up of the anammox process, substantially enhancing the aggregation and dynamics of the anammox consortium. Treating with 500 nM CA led to a 76.3% increase in anammox bacteria abundance compared to the control, resulting in exceptional nitrogen removal rates of 1.42 g N·L^−1^·d^−1^ within 54 days. This study unveils a mode of interspecies information transfer across trophic levels in aquatic organismal communities through CA-stimulated quorum sensing.

## Introduction

Quorum sensing (QS), a cell–cell communication process, relies on extracellular chemical signals known as autoinducers to orchestrate group behavior by triggering the transcription of specific genes [1–3]. QS involves signal release, signal recognition by specific receptors in the adjacent cells, and transcription regulation of the target genes by signal-bound receptors [1]. The collective behaviors of microorganisms orchestrated by QS encompass a variety of bacterial activities, including biofilm formation [4, 5], nitrogen metabolism [6, 7], toxin synthesis [8, 9], bioluminescence [10], bioaggregates development [11, 12], and modulation of mutation rates [13]. Those QS-regulated phenotypes primarily depend on the production of public goods, which are energetically expensive [14]. Therefore, based on the cooperative provision, QS-regulated behaviors are only advantageous to both individual bacteria and the group when a critical mass of individuals participates in activating the QS system [15–17]. The QS mechanism serves as a link in the broader social dynamics of microbial communities, facilitating the survival of mutants that do not respond to signals (acting as “social cheaters”), supporting *Vibrio fischeri* in its symbiotic relationship within the squid light organ, and enhancing bacterial anti-predator adaptations in response to *Caenorhabditis elegans* [17–19].

Microorganisms in both natural and engineered environments exhibit high phylogenetic diversity and exist in complex multi-species communities [20]. As the foundational organisms in many food webs, bacteria face predation pressure from eukaryotes, such as protozoa and nematodes. Studies have shown that bacterial attractants, such as isoamyl alcohol and diacetyl, along with QS autoinducers, can be detected by predators, thereby increasing the risk of predation [21–23]. In contrast, QS-mediated bacterial collective behaviors play a key ecological role in enhancing resistance to predation, including biofilm formation, the release of virulence factors, and morphological changes [18, 24, 25]. For instance, QS regulates biofilm formation in *Serratia marcescens*, promoting the development of filamentous, and chain-like cell structures to protect against protozoan predation [26]. For *Pseudomonas aeruginosa*, QS-mediated gene expression provides a selective advantage under heavy grazing pressure by promoting microcolony formation in early biofilms and increasing virulence factor production in later stages [27]. The continuous evolutionary arms race has driven bacteria to develop collective behaviors that enhance their survival in predator–prey interactions; however, the specific mechanisms by which bacteria detect and assess predation threats remain poorly understood. It is hypothesized that bacteria may rely on their promiscuous receptors to recognize chemical signals released by protozoa, thereby activating appropriate defense mechanisms via QS. A significant research gap persists in directly linking specific protozoan-secreted inducers to the QS system, underscoring the need for further investigation into these interactions’ molecular and ecological dynamics.

In artificial ecosystems, such as wastewater biotreatment systems, microbial community activities adapt to the development of functional bacterial communities and environmental factors, including pH, temperature, nutrient availability, redox potential, and pollutant concentrations [28–30]. QS-mediated cell consortia often facilitate biotreatment processes, particularly during the start-up stage of the granule sludge reactor and membrane bio-reactor [31, 32]. In the anaerobic ammonia oxidation (anammox) process, the cell density of anammox bacteria (AnAOB) morphotypes plays a critical role in process efficiency. This density-dependent regulation operates through HSL (N-acyl homoserine lactones)-mediated QS mechanisms, which facilitate microbial colonization by enhancing intercellular communication. The activated QS system strengthens the metabolic system’s resilience against environmental fluctuations and optimizes electron transfer efficiency within the microbial consortium through coordinated expression of redox-related proteins [33–35]. Effective regulation of QS development relies on exploring the mechanisms and types of interactions between signalling molecules and receptors. However, methods such as directly adding HSLs or extra-cultivating HSL-producing bacteria to enhance the QS require an extremely high investment. Therefore, exploring new inducers from predator–prey interactions, based on the social characteristics of microbial communities and the inclusiveness of HSL-mediated QS, is imperative and feasible.

In our preliminary experiments, we observed that the LuxR-type QS of *P. aeruginosa* PAO1 was affected by the secretions of *Paramecium caudatum.* This finding holds particular attraction given that *P. caudatum* serves as a natural predator of *P. aeruginosa* [36], and the latter has been extensively employed as a model organism for QS research due to its well-characterized QS signaling cascades [37, 38]. Motivated by the finding, this study identified cyanuric acid (CA) as a QS inducer of PAO1 from the *P. caudatum* secretions. To experimentally validate the ligand-receptor interaction, we engineered fluorescent reporter systems coupled with QS-deficient mutants of PAO1. This combinatorial approach demonstrated that CA functions as a dual agonist capable of activating canonical QS regulators—LasR (the transcriptional activator protein) and RhlR (HTH-type QS regulator). To elucidate the molecular basis of CA’s interaction with four LuxR-type regulatory factors, we employed molecular docking and molecular dynamics (MD) simulations. After the inducing ability to QS was confirmed, CA was applied to enhance the start-up of the anammox process. The addition of CA facilitated the maintenance of anammox activity under increasing nitrite stress, significantly boosting the abundance of AnAOB and thereby shortening the start-up duration. This study suggests that bacteria directly perceive chemical signals from protozoans, triggering the activation of QS, thereby highlighting the more significant ecological role of QS. The application of the newly discovered inducer in anammox provides valuable insights into the development of practical strategies that leverage QS inducers to enhance the stability, dynamics, and functional diversity of microbial communities, contributing to efficient wastewater remediation.

## Materials and methods

### Reconnaissance of QS inducer in *Paramecium* secretions

*P. caudatum* was cultured in a clean environment, and the secretions from mature *Paramecium* were harvested under sterile conditions. To evaluate the effect of *Paramecium* secretions on the QS signalling, *P. aeruginosa* PAO1 was cultivated in phosphate-buffered saline (PBS) containing a gradient proportion of *Paramecium* secretions. QS intensity of PAO1 was assessed by measuring the production of the 3OC12-HSL, which is a globally regulated cognate autoinducer [39, 40]. *Agrobacterium tumefaciens* KYC55 and *Chromobacterium violaceum* CV026 were employed to investigate the presence of HSLs in *Paramecium* secretions.

The next step is to search for potential QS inducers in *Paramecium* secretions. We analyzed pure *Paramecium* secretions, the culture filtrate of PAO1 (MS), and the filtrate of PAO1 cultured with 50% *Paramecium* secretions (denoted as PMS) using C18 reversed-phase HPLC–MS. By comparing the results, we could qualitatively determine the chemical compounds absorbed, assimilated, or decomposed from *Paramecium* secretions by PAO1. After verifying the stimulation effect of QS strength by using pure chemicals, we discovered that CA served as a potential inducer. Furthermore, the presence of CA in *Paramecium* secretions was quantitatively analyzed using LC–MS (Table S1).

The organic matter of *Paramecium* secretions and PAO1 secretion, influenced by *Paramecium* secretions and CA, was analyzed using 3D excitation-emission matrix (3D-EEM) fluorescence spectroscopy (F-7000, Hitachi, Japan).

Elastase, encoded by LasB and regulated by the LasR promoter, was employed to assess LasR activity in response to exogenous signalling molecules [41]. Changes in exopolysaccharide yield under CA-mediated QS were detected using the phenol–sulfuric method.

### Identification and mechanism of **CA** as a QS inducer

#### qPCR

To systematically evaluate the QS regulatory network, we performed quantitative PCR (qPCR) analyses targeting 18 key QS-related genes spanning multiple functional categories: (1) autoinducer synthases (*lasI, rhlI*); (2) transcriptional regulators (*lasR, rhlR, vfr, mvfR, ptxR*); (3) virulence factors (*lasA, lasB, toxA, aprA*); (4) phenazine biosynthesis components (*phzM*); (5) biofilm-associated genes (*pslA, pelA, algA*); and (6) rhamnolipid synthesis machinery (*rhlA, rhlB*). The tests were performed using the bacterial total RNA extraction kit (Sangon Biotech, CN). The PCR primers are shown in Table S2.

#### Construction of QS-deficient mutants and plasmid reporter systems

We used the homologous recombination gene method to knock out the *lasI* gene for 3OC12-HSL synthesis and the *rhlI* gene for C4-HSL synthesis in PAO1. PSim6-GmR was used to transform the competent PAO1 cells, and the strains with knocked-out genes were obtained by screening, sequencing, and expanded culture. Inspired by previous studies, a technique was developed to utilize the brightness of fluorescent protein as an indicator of the ligand-induced activity of exogenous LasR and RhlR [42, 43]. Plasmid reporter systems were assembled in *Escherichia coli*, an optimal host for expressing recombinant proteins, to assess the sensitivity of LasR and RhlR receptors to CA. Specifically, the isopropyl-beta-D-thiogalactopyranoside–induced *lasR*/*rhlR* was cloned on the PET plasmid, with the fluorescent protein (*pLasB-gfp*/*pRhlA-gfp*) downstream under the control of LasR/RhlR promoters. This methodology is fully detailed in the Supplementary Information.

#### Structural analysis

Molecular docking was conducted using AutoDock Vina (v4.2) to investigate the interactions between ligands (i.e. CA and cognate HSLs) and LuxR-type receptors. The 3D docking poses, ligand-protein interactions, and hydrogen bonds (H-bonds) in the docking complexes were visualized with Molecular Operating Environment V.2022, Discovery Studio Client V.19.0, and Pymol V.2.5.4, respectively. MD simulations were further performed using GROMACS (version 2022.4.1) to discern the conformational changes in the QS regulator upon interaction with ligands.

### Rapid start-up of anammox process

#### Anammox bioreactor

Four sequencing batch reactors (SBRs) with a working volume of 1 L each were used to examine the role of CA as a feasible QS inducer in expediting the start-up of anammox processes, with the influent NH_4_^+^-N and NO_2_^—^N concentrations of 240 and 200 mg/L, respectively. Here, NO_2_^—^N was set as the limiting substrate, and its consumption rate served as the indicator for adjusting the HRT [44]. Additionally, this influent concentration ensured sufficient acclimatization pressure and substrate supply for the AnAOB [45]. The dosage of CA was set to gradient 0 nM, 50 nM, 500 nM, and 5 μM according to the induction ability experiment in PAO1. The inoculum sludge comprised 10% finely dispersed mature anammox consortia, predominantly *Candidatus* Brocadia, and 90% anaerobic sludge from a secondary sedimentation tank at the Liede Wastewater Treatment Plant in Guangzhou. Because this experiment was designed as a short-term accelerated start-up test, the process gradually reduced hydraulic retention time (HRT), *i.e.* 12, 8, 6, and 4.8 h, across four phases within 60 days.

#### Chemical analysis

The analysis of NH_4_^+^-N, NO_2_^—^N, and NO_3_^—^N were measured daily according to the Chinese National Environmental Protection Standards (HJ535–2009, GB 7493–87, HJ/T 346–2007). The MLSS and MLVSS were determined following *Standard Methods for the*  *Examination of Water and Wastewater*, 22nd Edition [46]. Biomass samples were collected at the end of each phase to determine the protein and polysaccharide concentration in extracellular polymeric substances (EPS) and to analyze the particle size of the anammox consortia. Specific anammox activities were analyzed every 2 days using fresh biomass.

#### Biological analysis

The relative abundance of hydrazine synthase *β*-subunit (*hzsB*) of anammox bacteria, along with key functional genes associated with denitrification pathways—including a series of genes associated with denitrogenation capability nitrate reductase (*narG* and *napA*), nitrite reductase (*nirS* and *nirK*), and nitrous oxide reductase (*nosZ*) were quantified using qPCR. All seed sludge and the final anammox biomass in four SBRs were analyzed via high-throughput 16S rRNA gene amplicon sequencing, targeting the V3–V4 region with primers 338F/806R. PCR products were quantified, pooled, and clustered into OTUs at 97% similarity, with diversity analysis performed using the Majorbio Cloud platform.

#### HSL determination

The analysis of HSLs produced by the anammox biomass was conducted at the end of the start-up process. HSLs were extracted and concentrated from the mud-water mixture by ultrasonic waves. The extracted materials were analyzed using an LC–MS system, specifically the SCIEX QTRAP 4500 instrument.

### Facilitated rapid start-up of anammox under elevated stress by CA

Following confirmation of CA’s stimulatory effect on start-up process of anammox, an intensified operational strategy was implemented in a follow-up experiment. The experimental system incorporated parallel SBRs operating under phased hydraulic regulation: both control and CA-treated (500 nM) reactors underwent stepwise HRT reduction (16 h → 12 h → 8 h → 6 h), with modulation triggered exclusively by the treated reactor’s NO₂^—^N concentrations falling below 10 mg/L^−1^. Throughout the 35-day trial, inorganic nitrogen species in effluent were documented. Sludge samples were subjected to metagenomic analysis to elucidate microbial community dynamics and quantify functional genes associated with nitrogen metabolism, following Illumina’s standard protocol encompassing DNA extraction, library preparation, sequencing, and quality control. Clean reads were assembled and annotated using MEGAHIT, MetaGeneMark, and MMseqs2, while taxonomic composition and functional pathways were characterized via the BMKCloud platform. Detailed procedures are provided in the Supplementary Information.

### Statistical analysis

Statistical software, specifically Statistical Product and Service Solutions (SPSS), was employed to conduct an analysis of variance (ANOVA) on chemical analysis data. The purpose of this analysis was to ascertain the disparities between the bio-sludge treated with CA and the control that did not receive CA, using Dunnett’s post hoc test. The correlation between parameters was determined using the product moment correlation coefficient of the Pearson model. Non-metric multidimensional scaling was used to simplify the intricate nature of microbial community data. The evaluation of community similarity among all samples was conducted using the R statistical environment (version 3.2.2), employing the Bray–Curtis similarity index.

## Results and discussion

### CA is the effective constituent in *Paramecium* secretions

*P. aeruginosa* PAO1 (wild-type) is a typical QS strain, possessing an efficient LuxR-type QS system. The signalling circuit of HSL-mediated QS associated with PAO1 was characterized (Fig. S1). The QS systems in PAO1 operate in a hierarchical manner, with the *lasI/lasR* at the apex of the cascade, responsible for regulating the expression of the *pqs* and *rhl* genes [38]. As indicated by this illustrative QS network, 3OC12-HSL serves as a global autoinducer. We quantified its level to assess the influence of *Paramecium* secretions and the potential inducer they contain on the strength of the QS system. This part of the experiment was conducted at low cell density (OD600 = 0.5) to avoid superfluous autoinducers produced by crowded bacteria.

**Figure 1:**
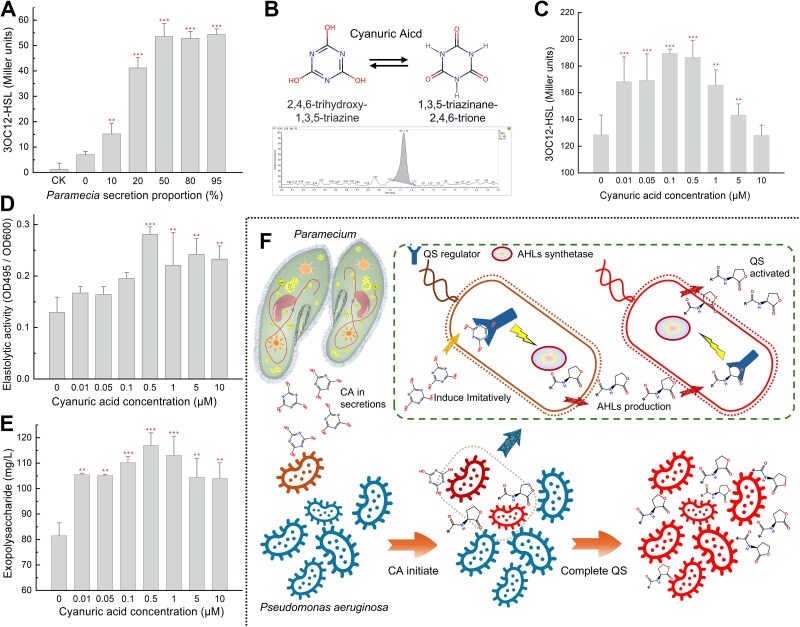
Cyanuric acid secreted from *Paramecium caudatum* induce QS in *Pseudomonas aeruginosa* PAO1. (A) The impact of varying doses of *Paramecium* secretions on the release of 3OC12-HSL by PAO1 in PBS. (B) The determination of CA in *Paramecium* secretions through LC–MS analysis. (C) The effect of varying concentrations of CA on the production of 3OC12-HSL by PAO1. (D, E) The effects of different doses of CA on the activities of elastase, exopolysaccharide production of PAO1. (F) The effect of CA released by *P. caudatum* on the quorum sensing of *P. aeruginosa* PAO1. One-way ANOVA with post hoc test by Dunnett’s multiple comparisons was conducted to compare the treatment and the control, where significant differences are indicated as follows: ^*^, *P* < 0.05; ^**^, *P* < 0.01; and ^***^, *P* < 0.001 (the same as following figures).

When cultured in PBS, the control PAO1 strain produced only 6.92 Miller units of 3OC12-HSL, indicating a weak QS strength. Upon the addition of *Paramecium* secretions, PAO1 showed enhanced QS strength, demonstrated by elevated 3OC12-HSL secretion. Supplementation with 50% *Paramecium* secretions resulted in a significant 7.72-fold increase in 3OC12-HSL secretion compared to the control (*P* < 0.001; Fig. 1A). The excessive production of 3OC12-HSL was theoretically attributed to enhanced QS either resulting from increased cell density or the effect of exogenous autoinducers. However, maintaining consistent cell density of PAO1 suggested that the nutrients in *Paramecium* secretions were insufficient for more bacterial reproduction (Fig. S2c). This observation indicated that there were some other QS signal molecules present in *Paramecium* secretions that stimulated the expression of *lasR*-regulated *lasI*. Furthermore, *A. tumefaciens* KYC55 and *C. violaceum* CV026 were employed to detect potential HSL components in *Paramecium* secretions; however, no one HSL autoinducer was identified. Consequently, it can be inferred that *Paramecium* secretions contains some uncovered QS inducers capable of effectively inducing LasR of PAO1. We compared the secretion composition of PAO1 with and without *Paramecium* secretions from the result of LC–MS. As such, CA (C_3_H_3_N_3_O_3_) was likely identified after ruling out the nutrients, toxins, metabolic wastes, and recalcitrant molecules according to their molecular formula and chemical composition (Fig. S2a). Subsequent LC–MS analysis revealed a quantified CA level of 1.78 μg/L in *Paramecium* secretions (Fig. 1B).

Biochemical validation is necessary to test the inducing of CA in QS regulation. The 0.01–10 μM CA range was set through alignment with endogenous QS molecule concentrations [47–50], while encompassing measured CA levels in *Paramecium* secretions (1.78 μg/L ≈ 13.8 nM). This setting ensures biological significance while maintaining ecological fidelity. Initially, the influence of CA was inferred based on its modulation of extracellular secretions, which is a characteristic change associated with QS regulation [51]. Analysis using 3D-EEM fluorescence showed higher levels of tryptophan proteins and humic acids in the EPS of PAO1 when exposed to 50% *Paramecium* secretions or 100 nM CA in PBS. This suggested that CA probably played a comparable role in inducing QS of PAO1 as *Paramecium* secretions (Fig. S2b).

To verify the inducing capability of CA, subsequent biological assays were conducted in 1% LB (Luria-Bertani) medium supplemented with CA. Adding CA at amount of 100 nM significantly enhanced 3OC12-HSL production in PAO1 by 51.0% (*P <* 0.05, Fig. 1C). Additionally, CA concentrations ranging from 10 nM to 10 μM elevated elastase activity, with 500 nM CA causing a substantial increase of 117.8% (*P <* 0.001, Fig. 1D). The inclusion of CA promoted the active secretion of extracellular polysaccharides, resulting in an increase of 28.1% to 43.5% (*P <* 0.001, Fig. 1E).

These findings indicate that CA, as an effective constituent secreted by *P. caudatum*, significantly modifies the QS of PAO1. Consequently, the potential signalling pathway for this inter-species communication was proposed (Fig. 1F). We hypothesized that the cognate HSLs associated with PAO1 could be replaced by CA. Thus, QS regulators within a subset of the PAO1 community were preemptively activated, leading to the concurrent synthesis of cognate HSLs, which in turn elicited QS signalling across most cell populations.

### LasR and RhlR respond to CA

To investigate the alteration in the gene expression associated with HSL-mediated QS in PAO1 upon CA addition, the transcription-quantitative PCR was employed. PAO1 was cultured in PBS containing 1%LB at a low cell density (OD 600 = 0.5). The results of gene content determination for PAO1 showed a significant response to CA. *lasR*, a pivotal QS regulator, exhibited significantly higher expression levels in the presence of 100 nM CA, being upregulated by 2.41-fold compared to the control. In line with *lasR* autoregulation, genes involved in elastase (*lasB*) and 3OC12-HSL synthesis (*lasI*) were upregulated by 2.51 and 2.71 times, respectively (Fig. S4). Similarly, we observed increased expression of *rhlI*, *rhlR*, and *rhlA*, which are involved in C4-HSL synthesis and mediation. Moreover, several other genes regulated by *lasR* also exhibited enhanced expression.

**Figure 2:**
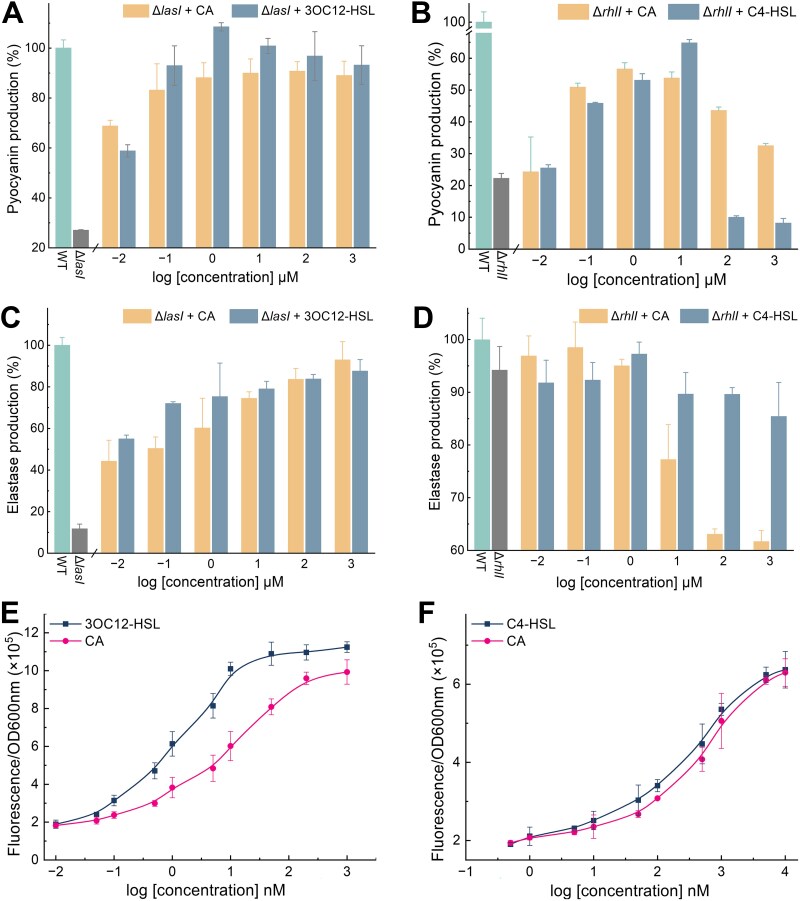
CA activates QS gene in deficient mutants and *E. coli* reporter strains. (A–D) The effects of different amounts of HSLs and CA on the relative expression of pyocyanin and elastase in QS-deficient mutants. (E, F) The fluorescence response of LasR in *E. coli* pLasB-gfp and RhlR in *E. coli* pRhlA-gfp to HSLs and CA. EC_50_ values were obtained by performing dose–response assays for each signal substance and analyzing the resulting data using nonlinear regression analysis.

The production of pyocyanin in PAO1, controlled by QS transcription factors, such as LasR, RhlR, and PqsR, was employed as a readout for the prediction of the QS-based communication [52, 53]. To elucidate the regulatory mechanism of QS signal transduction induced by CA in the absence of endogenous HSLs, we constructed the QS-deficient mutants, i.e. Δ*lasI* and Δ*rhlI* of PAO1, using the homologous recombination knockouts method. In PAO1-Δ*lasI*, without endogenous 3OC12-HSL synthesis, the pyocyanin production was decreased by 73.9% due to the lack of induction by active LasR in RhlI and RhlR. (Fig. 2A). Reversely, the secretion of pyocyanin was restored and reached its maximum value with the stimulation by adding 1 μM 3OC12-HSL or CA. However, excessive 3OC12-HSL addition hindered pyocyanin release, presumably due to receptor saturation or inefficient channel protein transmission. In PAO1-Δ*rhlI*, exogenous CA and C4-HSL at concentrations below 10 μM induced RhlR regulation and enhanced pyocyanin production (Fig. 2B). Excessive C4-HSL significantly suppressed RhlR activity, highlighting the importance of optimal autoinducer concentrations for RhlR activation [52]. The deletion of LasI markedly reduced the elastase production encoded by LasB, which was partially restored by introducing exogenous CA or 3OC12-HSL (Fig. 2C). Deleting *rhlI* had a minimal effect on the *lasB* function. Still, excessive CA or C4-HSL inhibited elastase production (Fig. 2D), implying that high CA levels in Δ*rhlI* strains could negatively impact the HSL-mediated QS system.

The plasmid reporter systems were constructed to investigate RhlR/LasR’s preference for CA. They assessed transcription from a LasR/RhlR-controlled promoter fused to a fluorescent protein (*pRhlA-gfp/pLasB-gfp*) in *E. coli*. LasR exhibited a pronounced preference for its cognate autoinducer 3OC12-HSL and CA, with EC_50_ values of 1.46 nM and 15.8 nM (Fig. 2E). In addition, C4-HSL and CA were identified as potent ligands for RhlR *pRhlA-gfp* reporter of *E. coli*, with EC_50_ values of 88.4 and 512.9 nM, respectively (Fig. 2F).

Based on these findings, we can confirm that CA is an effective inducer for the LuxR-type QS regulators, LasR and RhlR, in both PAO1 and engineered strains.

### The MD simulation verifies the preference for CA-induced QS

Many Gram-negative bacteria typically employ HSLs as autoinducers. LuxR-type proteins (such as LasR, transcriptional activator protein TraR, and regulatory protein SdiA), consisting of ligand-binding domains (LBD) and DNA-binding domains (DBD), represent a common class of HSL receptors, function as cytoplasmic transcription factors that bind with HSLs. LuxR-type receptors in diverse bacterial species may exhibit distinct preferences and specificities for HSLs [54]. LasR from *P. aeruginosa* can detect numerous long-chain HSLs [37]. TraR from *A. tumefaciens* selectively excludes any noncognate HSLs [55, 56]. SdiA from *E. coli* is highly promiscuous and vigorous in response to HSLs with different chain lengths [57, 58]. The varying affinities of these receptors with different signals enable flexible modulation of QS, which is critical for supporting signalling strength with competitive advantages over non-kin or antagonistic species. It aligns with current research on environmental microbiology, which indicates that increasing the concentration and variety of HSLs in the community benefits the early development of microbial treatment systems [34, 35, 59].

Structural alterations in the LBD of receptor proteins were investigated to better analyze the elusive interaction between the transcriptional regulatory factors and inducers of QS. Specifically, we elucidated the intricate mechanisms of the LBDs in LasR, RhlR, CviR, and TraR when bound to the primary HSL autoinducers and CA.

**Figure 3:**
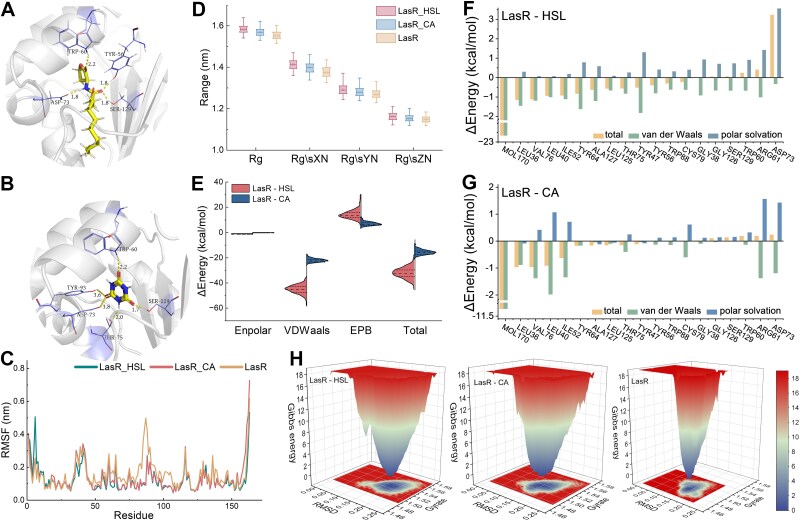
Hydrogen bonding and MD simulation analysis of LasR and RhlR. (A, B) The hydrogen bond between LasR and the 3OC12-HSL and CA. (C) RMSF of LasR and its ligand complexes observed during the MD simulation. (D) The changes in protein gyration radius during the receptor and complex simulation. (E) The binding energy of the complex through MMPBSA calculation. (F–G) Residue contribution in the calculation of binding free energy of MMPBSA. (H) The alterations in the free energy landscape of LasR and its ligand complexes observed during the MD simulation.

The high-affinity binding of CA to the receptors inside the ligand binding pocket was observed, attributed to its compact ring structure (Fig. S5a). In LasR, the HSL lactone ring formed four H-bonds with TYR56, TRP60, ASP73, TRP88, and SER129 (Fig. 3A, Fig. S5a). The residues TRP60 and SER129 formed similar H-bonds with CA, with bond lengths nearly equal to those with HSL (2.2 Å and 1.7 Å, 2.2 Å and 1.8 Å). ASP73 and TRP88 alternatively interacted through CA via anion-π and π-π stacking (Fig. 3B, Fig. S5a).

During the long MD simulation runs over 500 ns, the LasR-CA complex reached stability around 200 ns, as evidenced by Root Mean Square Deviation (RMSD) analysis (Fig. S9a). Observed distances between residues Tyr56—Asp73 and Ser129—Asp73 in LasR-CA fell between those of LasR-HSL and the empty protein (Fig. S6a-b). Root Mean Square Fluctuation (RMSF) analysis indicated that LasR-CA exhibited flexibility and mobility similar to LasR-HSL, demonstrating greater stability and rigidity compared to the empty protein (Fig. 3C). Radius of Gyration analysis revealed enhanced compaction of the LasR structure along the X-axis upon CA binding, like LasR-HSL, in contrast to the more expanded structure of the empty protein (Fig. 3D, Fig. S9a).

The binding free energies of the complex were calculated using the Molecular Mechanics Poisson-Boltzmann Surface Area (MMPBSA) method. The mean total binding energies (ΔGtotal) for HSL and CA binding to the LasR receptor were determined to be −32.4 and −15.9 kcal/mol, respectively. Specifically, the gas phase relative free energies (ΔGgas) were −45.3 and −22.5 kcal/mol, the solvation-free energies (ΔGsolv) were 12.9 and 6.5 kcal/mol, respectively (Fig. 3E). Both CA and HSL exhibited strong affinity for the hydrophobic ligand-binding pocket of LasR, with HSL exhibiting higher binding potential compared to CA. Energy analysis of residues further reinforced the vigorous affinity of both ligands (CA and HSL) with ASP73 and ARG61 in LasR, indicating increased protein hydrophobicity, which aids in protein assembly and dimer formation [60] (Fig. 3F, G). In both LasR-HSL and LasR-CA complexes, binding was primarily mediated through van der Waals interactions with residues LEU36, VAL76, and LEU40. Additionally, HSL interacted with ILE52 and CA interacted with ARG61 and ASP73. Analysis of the short-range Lennard-Jones potential (LJ-SR) revealed that electrostatic repulsion between the empty LasR protein and the aqueous solvent was more pronounced than the LasR-HSL and LasR-CA complexes (Fig. S6c). Principal Component Analysis (PCA) of MD simulation trajectories, coupled with Free Energy Landscape (FEL) analysis, offered insights into the energy distribution in protein-ligand interactions [61]. Unlike empty protein, LasR-HSL and LasR-CA exhibited conformational transformation paths and the minimum energy states.

The interactions between the transcriptional regulator RhlR and ligands (its cognate autoinducer C4-HSL and CA) were thoroughly scrutinized. Molecular docking and MD simulation showed that CA forms stable H-bonds with residues TRP68 and ASP81, resulting in a strong binding energy of −16.6 kcal/mol for the RhlR complex. This complex displays a conformational change pattern similar to that observed in the RhlR-HSL complex (Fig. S7).

CviR and TraR, LuxR-type QS transcriptional regulators found in *C. violaceum* and *Agrobacterium*, were examined to investigate the promiscuity of CA. The terminal methyl of HSL established a robust π-sigma bond with TYR88 in CviR (Fig. S5c), a feature not observed in the LasR and RhlR complexes. This indicates that the ligand binding pocket of CviR is specifically designed for the linear conformation of its active ligand. During the docking of CA with TraR, a significant repulsive force caused by a negative charge between ligand and receptor was detected, resulting in the temporary ejection of CA from the binding pocket during MD simulation (Fig. S5d). Additionally, the TraR-CA complex exhibited significant structural instability during MD simulations, contrasting sharply with the stability observed in the TraR-HSL complex.

The absence of robust bonds (π-sigma bond in CviR-HSL) or the interposition of repulsive forces (unfavorable negative–negative interactions in TraR-CA) suggested that CA loses the ability to bind effectively with both CviR or TraR. This finding was reinforced by the biological assays conducted with CV026 and KYC55, where no QS signalling activity of CA or *Paramecium* secretions was detected. In contrast, the LasR protein features a distinctive and flexible circular pocket, endowing it with lenient specificity and promiscuity in ligand sensing [62]. Consequently, CA is inclined to bind LasR-LBD, similar to the way the lactone ring of HSLs interacts with LasR-LBD. These interactions play an indispensable role in the early QS initiation regulated by LasR. Likewise, RhlR is prone to accommodate CA as an effective ligand [63].

Cyanuric acid, an intercellular chemical signal released by the predator *Paramecium*, is detected by QS bacteria like PAO1, which can activate the LuxR-QS system even at low cell densities. The activation of QS in PAO1 enhances biofilm formation and toxin secretion, conferring advantages for resisting predation and establishing a more stable ecological niche [64, 65]. The induction of QS by CA is achieved through active coordination with promiscuous receptors such as LasR and RhlR in PAO1. The flexible ring structures near the ligand-binding sites of these receptors allow for the accommodation of certain ligand promiscuity [62, 66]. The lactone ring of HSLs can be replaced by the hexagonal ring of CA, forming robust H-bonds or other hydrophobic interactions with amino acid residues in LBD. The two structures of CA (triphenol and trione) enhance ligand-receptor binding by providing polar hydrogen atoms. This finding provides a perspective into the interactions between interspecies across different trophic levels, suggesting that QS can serve not only as a communication system among bacteria but also as part of a comprehensive “Internet” crisscross microorganism in the aquatic environment.

### CA accelerates the start-up of the anammox process

The preceding sections of this study demonstrate that CA serves as a stimulatory inducer, substituting for HSL in specific LuxR-type transcriptional regulators, including LasR and RhlR. During the start-up of the anammox process, HSLs-mediated LuxR-type QS has been documented to be essential for microbial aggregation, helping microbes adapt to stressful environments and promoting the abundance of AnAOB [45, 67]. In the subsequent section, we investigated the effect of CA on accelerating the start-up process in anammox.

**Figure 4:**
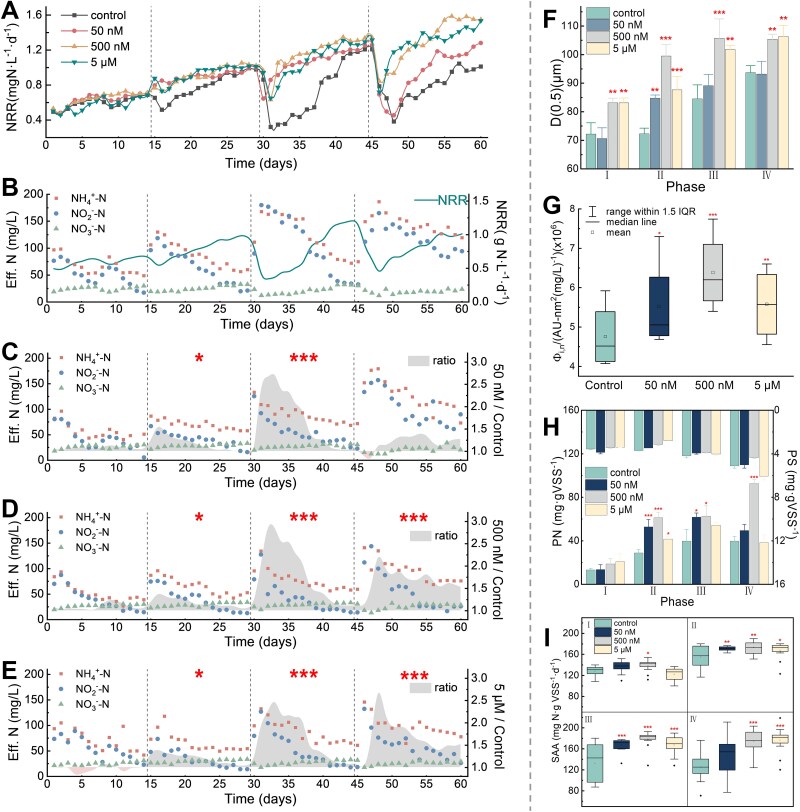
Cyanuric acid expedited the start-up of the Anammox process. (A) The NRR of the control and the treatment over 60 days. (B) The concentrations of different nitrogen species in the effluent of the control. (C–E) The concentrations of different nitrogen species and corresponding NRR in the control and the treatment. (F) The effect of CA on particle size distribution at the end of each phase. (G) Fluorescence regional integration (FRI) on EEM of extracellular secretion in the IV region. (H) The extracellular secretory polysaccharide (PS) and extracellular secretory protein (PN) of anammox sludge. (I) the specific anammox activity (SAA) of sludge in each phase.

During Phase I of the start-up process, with an HRT of 12 h, reactors treated with 50 nM and 500 nM CA exhibited a minor improvement in denitrification efficiency (Fig. 4A). By the end of Phase I, nitrogen removal rates (NRR) across all reactors ranged from 0.69 to 0.71 gN·L^−1^·d^−1^, displaying comparable level. Throughout Phase II, operating at an HRT of 8 h, all groups encountered a transient reduction in denitrification performance attributable to fluctuating and stressful conditions induced by nitrite toxicity. However, once AnAOB adapted to the increased nitrogen loading, anammox efficiency gradually improved, with CA-treated reactors recovering more quickly than the control. In Phase III, the average NRR of the three CA treatments was enhanced by 42.8%, 56.1%, and 45.7% compared to the control, respectively (*P <* 0.001, Fig. 4C–E), highlighting the significant role of CA played in restoring anammox function. NRR of 1.4 gN·L^−1^·d^−1^ was achieved within 54 and 57 days for the treatments with 500 nM and 5 μM CA, respectively. This finding indicated that CA significantly aided the start-up of an anammox reactor. However, under the high HRT load, both the control group and the treatment with only 50 nM CA struggled to recover effectively by the end of the process.

A correlation (*R*^2^ = 0.63, 0.56, 0.79, and 0.80) was found between effluent pH and NRR, primarily attributed to the sustained production of alkaline substances during the anammox processes [68] (Fig. S11a). Treatments with 500 nM and 5 μM CA demonstrated significantly larger particle sizes (*P <* 0.01, Fig. 4F), suggesting that the presence of CA positively influenced the granulation of anammox sludge. Furthermore, the EEM spectra of extracellular secretions from anammox sludge indicated that the primary peaks fell within Region IV, with excitation and emission wavelengths between 250 and 380 nm (Fig. S4). This region is commonly associated with secondary substances derived from soluble proteins, specifically aromatic amino acids such as tryptophan or tyrosine (Fig. S10). Using Raleigh and Raman scattering reduction techniques, we quantified the fluorescence intensity of the peaks in Region IV (Fig. 4G). This analysis showed that the addition of 500 nM CA markedly enhanced the production of extracellular secretions (*P* < 0.001). The quantitative chemical composition analysis indicated that extracellular protein (PN) constituted the dominant component of these secretions (Fig. 4H). At the end of Phase II, the PN levels in treatments with 50 nM, 500 nM, and 5 μ M CA showed significant increases of 83.4%, 125.2%, and 44.3%, respectively, compared to the control (*P* < 0.05). The addition of 500 nM CA resulted in an 11.7% rise in specific anammox activities during Phase II (*P* < 0.01). As HRT loading escalated in Phase III, a significant decline was observed in the control group’s performance (Fig. 4I). In contrast, the treatment with 500 nM CA exhibited a notable 35.6% enhancement in anammox efficiency, reaching 179 mgN·gVSS-1·d-1 (*P* < 0.001). By Phase IV, while the control recorded a decrease to 125 mgN·gVSS^−1^·d^−1^, reactors treated with 500 nM and 5 μM CA maintained higher activity levels, recording improvements of 39.0% and 40.9% over the control, respectively (*P <* 0.001). Overall, these findings demonstrate that CA significantly improved the anammox activity of bacterial consortia, particularly under conditions of excessive influent loading.

### CA improves the evolution of anammox biomass

**Figure 5:**
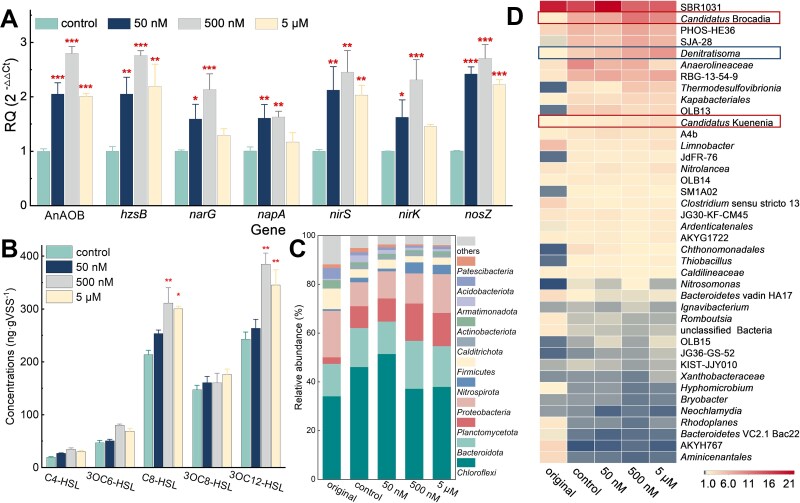
CA enhances the development of the anammox colony by facilitating QS. (A) The effect of CA key on the relative gene expression associated with nitrogen metabolism based on qPCR analysis. (B) The effect of CA on the secretion of HSLs. (C, D) The effect of CA on the secretion of HSLs. The effect of CA on changes in the structure of sludge microbial community at the phylum and genus level.

To investigate gene expression related to the anammox process and specific nitrogen metabolism, we utilized transcription-qPCR and analyzed the data using the Livak method (Fig. 5A). The 500 nM CA treatment resulted in the highest levels of gene overexpression, with expression multiples of 2.76 for *hzsB*, 2.13 for *narG*, 1.63 for *napA*, 2.45 for *nirS*, 2.31 for *nirK*, and 2.71 for *nosZ*. These results suggest that CA significantly contributed to the establishment of a dynamic and complex denitrogenation network.

We employed high-throughput sequencing technology to assess the impact of CA on the microbial community structure (Fig. S11). We observed a reduction in bacterial diversity across all reactors, with 503 species absent at the end of the process, indicating the emergence of specialized microbial communities (Fig. S12a). *Planctomycetota*, a phylum that encompasses major AnAOB, constituted only 2.7% of the total microbial population in the seed sludge (Fig. 5C). After 60 days, its relative abundance inthecontrol significantly increased to 8.6%. In the CA treatments, this abundance rose to 9.5%, 15.3%, and 13.6%, respectively. At the genus level, *Candidatus* Brocadia*,* a typical AnAOB, made up 6.8% of the total population in the control. This proportion increased by 1.71 times with the 500 nM CA treatment and 1.44 times with the 5 μM CA treatment compared to the control. The relative abundance of *Candidatus* Kuenenia also increased to 1.4%, 2.3%, and 2.7% in the 50 nM, 500 nM, and 5 μM CA treatments, respectively, compared to 1.1% in the control (Fig. 5D). These findings indicate that the addition of CA significantly improved the adaptability of AnAOB to unfavorable fluctuation, aiding the recovery of reproductive activity in anammox consortia from inhibition during the start-up process.

To evaluate the crucial role of HSL-mediated QS in AnAOB that accelerates anammox start-up processes triggered by CA, we selected five representative HSLs for quantification analysis, based on previously identified autoinducers in the anammox literature [45, 69, 70] (Fig. 5B). The most predominant autoinducer was determined to be 3OC12-HSL, followed by C8-HSL. The addition of 500 nM and 5 μM CA strikingly increased the production of C8-HSL (45.5% for 500 nM; 40.5% for 5 μM, *P* < 0.05) and 3OC12-HSL (40.5% for 500 nM; 42.2% for 5 μM, *P* < 0.01), respectively. This indicates a significant improvement in the strength of HSL-mediated QS in the presence of CA.

The activity of AnAOB was affected by nitrite toxicity, which is considered the most noticeable inhibitor in the anammox processes. The half-inhibitory concentration (IC_50_) value for NO_2_^—^N in the anammox process was reported to be 50 mg/L [71]. The NRR decreased with increasing influent loading due to excessive nitrite at the beginning of each phase. The addition of CA enhanced the extracellular secretion of microorganisms and increased sludge particle size. These changes improved resistance against NO_2_^−^ toxicity and aided in the recovery of anammox activity.

### Quick start-up of anammox under elevated pressure

**Figure 6:**
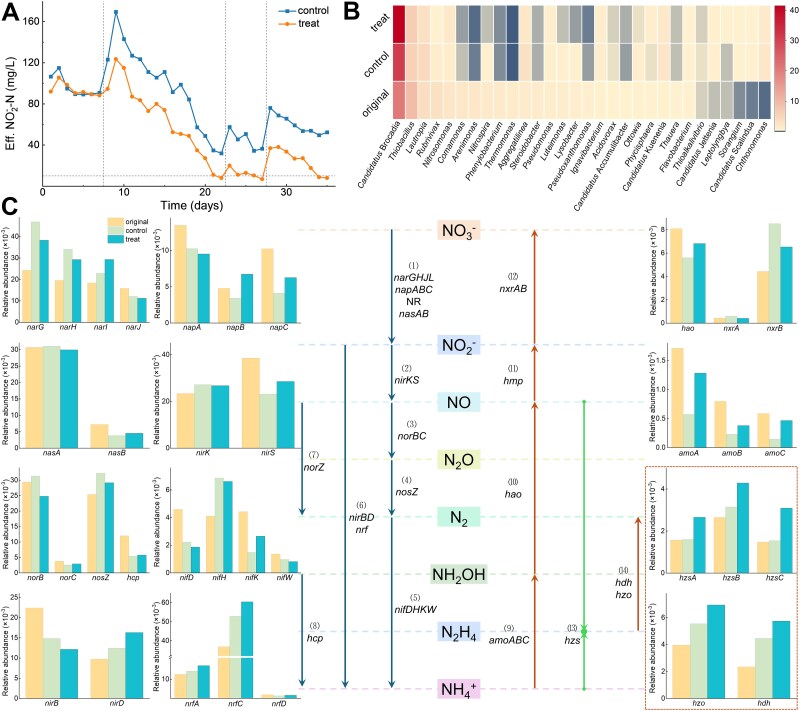
Facilitated rapid start-up of Anammox under elevated stress by CA. (A) The concentrate of NO_2_^—^N in the effluent of the control and the treatment over 35 days. (B) The effect of CA on changes in the structure of sludge microbial community at genus level. (C) The changes of gene abundance of inorganic nitrogen metabolism.

Under the intensive stress strategy, the addition of 500 nM CA significantly enhanced the rapid start-up of the anammox process. The experimental group consistently demonstrated superior NO_2_^−^ removal efficiency compared to the control group at each operational phase (Fig. 6A). This optimization achieved a remarkable nitrogen removal rate (NRR) of 1.41 mgN·L^−1^·d^−1^ within 35 days. In the genus-level statistics derived from the metagenome analysis, the key anammox bacterium, *Candidatus* Brocadia, increased from 20.8% in the seed sludge to 41.3% in the CA-treated reactor, compared to 31.1% in the control (Fig. 6B). At a more specific species level, *Candidatus* Brocadia sinica, the dominant species, exhibited a proliferation rate 1.32 times greater in the CA-treated than in the control (Fig. S13c). The abundance of *Candidatus* Brocadia sp. BROELEC01 was 0.94% in the seed sludge, but it decreased to 0.79% in the control after the start-up, indicating a negative response to the rapidly increased HRT load. In contrast, in the CA-treated, this species experienced mitigated stress effects, with its abundance rising to 2.02%. Furthermore, the introduction of CA slowed down the rapid proliferation of other species such as *Chlorobi bacterium OLB4*, *Proteobacteria bacterium*, and *Ignavibacteriae bacterium* observed in the control, thus contributing to the stability of the system. These findings collectively demonstrate CA’s stimulatory effect on AnAOB enrichment during start-up process.

Concomitant microbial shifts were observed: *Proteobacteria* (dominated by Thiobacillus) experienced marked decline during acclimatization. Whereas the *Chloroflexi* phylum demonstrated a significant increase in proliferation—from 13.2% to 22.3% in the control (Fig. S13b), supplementation with 500 nM CA further enhanced this proliferation to 30.5%. Recognized as critical components in various anammox systems, *Chloroflexi* are heterotrophic organisms capable of utilizing organic carbon derived from deceased microorganisms [72, 73]. This increased proliferation likely reflects intensified restructuring of the microbial community under CA exposure, with *Chloroflexi* capitalizing on materials from lysed cells due to population turnover. The *Anaerolineae* among then, confirmed to possess denitrification genes and participate in the dissimilatory nitrate reduction to ammonium (DNRA) processes [74–76], align with the observed upregulation of the *nrfA* gene. The synchronized proliferation of Chloroflexi and AnAOB suggests potential functional complementarity, possibly through the cross-feeding of DNRA-derived nitrogen. The addition of CA not only enhanced biomass accumulation but also fostered cooperative interactions. This ecological synergy offers new perspectives for engineering DNRA-anammox coupled systems to enhance nitrogen removal efficiency.

Comparative metagenome analysis revealed critical insights into nitrogen metabolic pathways. The *hzs* gene cluster (encoding hydrazine synthase) showed pronounced upregulation in the CA group, with *hzsC* subunit abundance increasing by 109.2% compared to merely 4.6% in the control. Similarly, *hzo/hdh* genes (catalyzing hydrazine oxidation to N₂) exhibited 75.1% and 145.0% enrichment in the experimental system, respectively, surpassing control group increments of 40.0% and 90.3%. These genetic patterns substantiate CA’s positive regulation on core anammox metabolic pathways.

Previous studies have demonstrated the efficacy of direct supplementation with HSLs or HSL-enriched supernatant in accelerating anammox reactor start-up [77–79]. Current evidence suggests that most HSLs positively influence density-dependent anammox consortia primarily through stimulating EPS biosynthesis. However, microbial reactors constitute complex ecosystems where AnAOB face substrate competition from coexisting nitrogen-cycling populations. Exogenous C12-HSL supplementation has been shown to significantly stimulate the proliferation of heterotrophic bacteria and enhance denitrification processes [67], potentially creating nitrite-limiting conditions that compromise anammox performance. Whereas specific HSLs like C6-HSL and 3OC6-HSL can orchestrate intercellular cooperation to establish collaborative communities, such artificial modulation inevitably increases ecological instability in anammox systems [79]. These surveys underscore the critical importance of our identifying QS-inducer-responsive populations (*Candidatus* Brocadia and *Anaerolineae*) for targeted manipulation of functionally relevant microbial consortia. The observed QS-mediated dynamic community succession may provide ecological advantages through dual synergistic mechanisms: facilitating environmental adaptability by enriching stress-resistant genotypes under fluctuating conditions [80] while concurrently forging cross-species defensive networks through microbial interactions [81, 82]. This ecological strategy aligns with our finding that CA-induced QS reinforcement enhances AnAOB resilience against nitrite stress through improved anammox activity. QS microorganisms construct complex interaction networks in bioreactors, and directional enhancement of biological treatment based on HSLs still requires more theoretical and practical support. The research on the inducer CA introduced a branch for the technical system of QS-enhanced biological treatment, providing inspiration for future research based on cross-trophic QS regulation.

The QS activity and engineering value of CA are confirmed, which is also important for its environmental and ecological discussion. CA has served as a crucial industrial chemical since its initial discovery and utilization in the 19th century [83]. Industrial synthesis of CA primarily involves urea pyrolysis at 250°C. As a key chemical precursor, CA finds extensive applications in manufacturing trichloroisocyanuric acid disinfectants [83, 84], triazine herbicides [85], and flame retardants [86]. Subsequent environmental release of these products introduces CA through metabolic intermediates, with melamine—another widely used s-triazine compound constituting an additional primary environmental source [87]. Environmental monitoring reveals CA’s ubiquitous presence in both terrestrial and aquatic systems. Chinese surface soils contain CA at concentrations ranging from 3.68 to 211 ng/g (median 17.0 ng/g) [87]. New York State water surveys demonstrate substantial CA levels across various water bodies [88]: swimming pool water exhibits exceptionally high concentrations (median 1.5 × 10^7^ ng/L), followed sequentially by precipitation (650 ng/L), wastewater (608 ng/L), tap water (316 ng/L), seawater (147 ng/L), lake water (96 ng/L), river water (81 ng/L), and bottled water (56 ng/L). CA demonstrates notable resistance to photocatalytic degradation [89], with environmental breakdown primarily mediated by CA hydrolases [90]. Key enzymes include TrzD from *Acidovorax citrulli* strain 12227, AtzD from *Pseudomonas sp*. strain ADP, and CAH from *Moorella thermoacetica* ATCC 39073, whose catalytic pathways are illustrated (Fig. S3). In addition, certain fungi utilize CA as a nitrogen source [91, 92] too. Bacterial cyanurate hydrolase genes predate industrial s-triazine production by ~150 years [91, 93–96], suggesting that CA might have existed in nature long before. Phylogenetic analyses further support the ancient evolutionary origin of these catabolic genes [97]. Intriguingly, the biosynthetic origins of environmental CA remain poorly understood, with the LOTUs database containing the sole documented reference to potential biological synthesis [98]. Our cultivation experiments with *Brachionus calyciflorus* rotifers showed no detectable CA secretion (detection limit: 10 ng/L) (Table S6). Environmental monitoring revealed trace levels of CA (56 ± 14 ng/L) in wastewater influent, whereas significantly higher concentrations (266 ± 81 ng/L) were detected in Guangzhou tap water. The highest CA concentration was recorded from the surface water of the Pearl River (2870 ± 790 ng/L), which may be due to the pollution from surface runoff and the abundant metabolism and secretion of *Paramecium* and other microorganisms. Effluent from the anammox reactor (Section 3.6) was analyzed: the control group showed no detectable CA (<10 ng/L), while the 500 nM CA-treated group retained only 972 ± 47 ng/L of CA (66 ± 3.2 mg/L). It means that 98.5% of supplementary CA was degraded, which aligns with microbial CA degradation mechanisms in anoxic environments [99, 100]. The observed low CA levels in wastewater influent reflect pre-degradation during anaerobic fermentation in sewer networks prior to plant entry.

The dual origin of CA as both an industrial contaminant and a potential natural product underscores the critical need to elucidate its QS induction mechanisms. Such exploration holds profound implications for ecological stability and microbial community dynamics.

## Conclusion

This study reveals that CA, a metabolite from *P. caudatum*, serves as a potent QS inducer by activating LuxR-type regulators in *P. aeruginosa*, thereby facilitating interspecies communication across trophic levels. The CA-mediated QS activation enhances microbial aggregation, biofilm formation, and stress resilience, which accelerates the start-up of the anammox process by optimizing microbial community dynamics and functional stability. These findings underscore the ecological significance of predator–prey chemical interactions in modulating microbial consortia and highlight CA’s potential as an eco-engineering tool to bolster wastewater treatment efficiency. By bridging microbial social behavior and ecological function, this work advances strategies for harnessing natural signaling molecules to engineer robust biotechnological systems.

## Supplementary Material

Supplementary_Information_wraf080

## Data Availability

The raw 16S rRNA gene amplicon sequencing and metagenome data are available in the Sequence Read Archive under BioProject accession PRJNA1246756. All the other data supporting the conclusions of this study are available in the paper and supplemental materials.
