# Supplementary material for: Cyanuric acid in Paramecium secretions is an efficient quorum sensing inducer
Source: ISME J. 2025 Aug 8;19(1):wraf080. doi: 10.1093/ismejo/wraf080 (PMC12342508; doi:10.1093/ismejo/wraf080)
Supplement: Supplementary_Information_wraf080 [file supplementary_information_wraf080.doc]

**Supplementary Information**

**For**

**Cyanuric Acid in Paramecium Secretions**

**is an Efficient Quorum Sensing Inducer**

*Xingyao Ye ^a, b^*, *Xiaojun Niu* *^a, b, c, *^*, *Dongqing Zhang* *^b, *^*, *Mengyu Lv ^a^*, *Ling Li ^a^*, *Qiang Liu ^a^*, *Deye Chen ^d^, Yu Lin ^e^, Zhiquan Yang ^a^, Yi Zhang ^a^*

a School of Environment and Energy, South China University of Technology, Guangzhou, 510006, PR China

b School of Environmental Science and Engineering, Guangdong University of Petrochemical Technology, Maoming, 525000, PR China

c The Key Lab of Pollution Control and Ecosystem Restoration in Industry Clusters, Ministry of Education, South China University of Technology, Guangzhou Higher Education Mega Centre, Guangzhou, 510006, PR China[[1]](#footnote-2) 

d China Water Resources Pearl River Planning Surveying and Designing Co. Ltd. Guangzhou 510640, PR China

e Guangzhou Urban Drainage Company Limited, Guangzhou 510006, PR China

**CONTENT**

[Text S1. Culture of *paramecium* and collection of PCS. 4](#__RefHeading___Toc196410080)

[Text S2. PSC effect on 3OC12-HSL production of *Pseudomonas aeruginosa* PAO1 4](#__RefHeading___Toc196410081)

[Text S3. Biological examination of HSLs. 5](#__RefHeading___Toc196410082)

[Text S4. LC-MS analysis of PCS 6](#__RefHeading___Toc196410083)

[Text S5. Quantification of cyanuric acid 7](#__RefHeading___Toc196410084)

[Text S6: Elastase Assay 8](#__RefHeading___Toc196410085)

[Text S7: Exopolysaccharide Assay 8](#__RefHeading___Toc196410086)

[Text S8: QPCR of PAO1 9](#__RefHeading___Toc196410087)

[Text S9: Construction of *P. aeruginosa* PAO1-ΔLasI/ΔRhlI 9](#__RefHeading___Toc196410088)

[Text S10: Construction of fluorescent plasmid reporter strain 10](#__RefHeading___Toc196410089)

[Text S11: Molecular docking 10](#__RefHeading___Toc196410090)

[Text S12: Molecular dynamics simulation 11](#__RefHeading___Toc196410091)

[Text S13: Anammox bioreactor 12](#__RefHeading___Toc196410092)

[Text S14: Chemical analysis method 13](#__RefHeading___Toc196410093)

[Text S15: qPCR of AnAOB genes 14](#__RefHeading___Toc196410094)

[Text S16: Community diversity of anammox 15](#__RefHeading___Toc196410095)

[Text S17: HSL quantification in AnAOB 15](#__RefHeading___Toc196410096)

[Text S18: Metagenome of anammox under high pressure start-up 16](#__RefHeading___Toc196410097)

[Text S19: MD analyses of C4-HSL and CA induce RhlR 17](#__RefHeading___Toc196410098)

[Figure S1: The HSL-mediated group sensing pathway in *Pseudomonas aeruginosa*. 19](#__RefHeading___Toc196410099)

[Figure S2: Analysis of PCS and impact of CA on PAO1 growth 19](#__RefHeading___Toc196410100)

[Figure S3: Source and degradation of CA 20](#__RefHeading___Toc196410101)

[Figure S4: CA affects the QS gene expression in PAO1 21](#__RefHeading___Toc196410102)

[Figure S4: Molecular docking and bonding 21](#__RefHeading___Toc196410103)

[Figure S6: The distance between important pairs of residues and LJ-SR of receptors. 22](#__RefHeading___Toc196410104)

[Figure S7: MD simulation analysis of RhlR 23](#__RefHeading___Toc196410105)

[Figure S8: Hydrogen bonding and MD simulation analysis of CviR and TraR 24](#__RefHeading___Toc196410106)

[Figure S9: Molecular dynamics simulation analysis 26](#__RefHeading___Toc196410107)

[Figure S10: 3D-EEM of extracellular secretion of AnAOB 27](#__RefHeading___Toc196410108)

[Figure S11: Alteration of pH, MLSS and MLVSS 28](#__RefHeading___Toc196410109)

[Figure S12: Species diversity analysis of AnAOB 28](#__RefHeading___Toc196410110)

[Figure S13: Quick Start-up of Anammox under High Pressure 29](#__RefHeading___Toc196410111)

[Table S1: Mass spectrometry parameters of cyanuric acid quantification 30](#__RefHeading___Toc196410112)

[Table S2: Primer used in this study 30](#__RefHeading___Toc196410113)

[Table S3: The formula of inlet water 32](#__RefHeading___Toc196410114)

[Table S4: The basic properties of HSLs. 33](#__RefHeading___Toc196410115)

[Table S5: Parameters for the procedure of gradient elution. 33](#__RefHeading___Toc196410116)

[Table S6: The concentration of CA in water sample 33](#__RefHeading___Toc196410117)

## Text S1. Culture of *paramecium* and collection of PCS.

The process began by boiling 10g of dried straw in 1L of deionized water for 20 minutes. The resulting mixture was filtered using qualitative filter paper to produce a transparent culture medium. *Bacillus subtilis* spores were able to thrive and multiply in this liquid, providing nourishment for *Paramecium*. Using a syringe, 50 *Paramecium* individuals were extracted from the seed solution and introduced into each liter of the culture solution at 25°C. After three days, the population density of the *Paramecium* reached approximately 500 ind/mL. The *Paramecium* were obtained by filtration using a 400-grit sieve and subsequently rinsed ten times with sterile water to eliminate contaminants and bacteria. The *Paramecium* were then placed in sterile deionized water at a concentration of 1000 ind/mL for 12 hours to release their secretions. The mixture was filtered using gauze with a mesh size of 400, followed by filter papers with pore sizes of 1μm, 0.45μm, 0.22μm, and 0.1μm, respectively. This process resulted in obtaining a pure and sterile secretion filtrate (PCS). All containers were sterilized at 121°C, and the operations were carried out on a vertical flow clean bench.

## Text S2. PSC effect on 3OC12-HSL production of *Pseudomonas aeruginosa* PAO1

The *P. aeruginosa* PAO1 strain, preserved in 35% glycerol, was inoculated into Luria-Bertani (LB) broth with a 2% inoculum ratio. The culture was incubated at 37°C with 120 rpm for 16 hours until reaching the stationary phase. The bacterial culture underwent three centrifugation cycles at 6,000 rpm, followed by washing with sterile phosphate-buffered saline (PBS) at pH 7.2. The PCS was mixed with sterile PBS in various proportions, and the collected PAO1 cells were cultured at 37°C for 12 hours after centrifugation. The optical density at 600 nm (OD600) was measured pre- and post-experimentation. The quorum sensing signaling in *P. aeruginosa* was determined by the amount of 3OC12-HSL produced. The concentration of 3OC12-HSL was assessed using the strain *Agrobacterium tumefaciens* KYC55.

## Text S3. Biological examination of HSLs.

The concentration of long-chain HSL can be assessed using the strain *Agrobacterium tumefaciens* KYC55 with high detection efficiency. A 1% inoculum of KYC55, preserved in 35% glycerol, was cultured in AT medium (supplemented with tetracycline, kanamycin, and spectinomycin) for 8 hours at 30°C. The test solution and a 1% DMSO solution were added to the growth medium at a 2% proportion after being filtered through a 0.22μm membrane. β-galactosidase activity was measured after a 6-hour incubation. Briefly, 1 mL of KYC55^1^ culture was added to 2 mL of Z buffer (0.06 M Na_2_HPO_4_, 0.04 M NaH_2_PO_4_, 0.01 M KCl, 0.001 M MgSO_4_·7H_2_O, 0.05 M β-mercaptoethanol, pH 7.0). Cells were permeabilized by adding 100 μL of 0.1% SDS and 200 μL of chloroform. Subsequently, 200 μL of 4 mg/mL ONPG (o-nitrophenyl-β-D-galactopyranoside) was added to the mixture. The reaction was terminated by adding 200 μL of 1 M Na_2_CO_3_ after 10 minutes in a 50°C water bath. Cell debris was removed by centrifugation at 14,000 × g for 30 seconds, and the supernatant absorbance was measured at 420 nm using a Shimadzu UV-2700 spectrophotometer. β-galactosidase activity was expressed in Miller units (MU) and calculated as follows: 1,000 × OD420 / (T [min] × V [mL] × OD600).

20× AT buffer: 214 g KH_2_PO4, 1 L distilled water, KOH solution to adjust the pH to 7.3.

20× AT salt solution: 0.044 g MnSO_4_·H_2_O, 0.1 g FeSO_4_·7H_2_O, 0.152 g CaCl_2_, 1.56 g MgSO_4_, 40 g NH_4_HSO_4_, 1 L distilled water.

AT culture medium: 50 mL of 20× AT salt solution, 50 mL of 20× AT buffer solution, 10 mL of 50% (w/v) glucose solution, and 890 mL of distilled water. Each component was sterilized separately, with the AT salt solution, AT buffer solution, and distilled water sterilized at 121°C for 20 minutes under high temperature and pressure, and the glucose solution sterilized using a 0.22μm filter membrane.

The short-chain HSL was examined by *Chromobacterium violaceum* CV026.

Culture the Bacteria: Incubate the bacteria to be detected in the culture medium at 37°C for 6, 12, 18, 24, 30, 36, and 42 hours.

Centrifuge and Prepare Supernatant: Centrifuge the cultures at 10,000 rpm for 10 minutes. Collect the supernatant and store it for later use.

Prepare LB Agar Plates: Add 1% agar to LB medium for sterilization. Then, add 5 ml of activated reporter Bacillus CV026 to 50 ml of LB agar, mix well, and pour the mixture into plates.

Create Wells and Add Supernatant: After the agar solidifies, punch a hole in the center of each plate. Add the culture supernatant of the bacteria to be detected into the hole. Incubate the plates at 28°C for 24 hours.

Observe and Measure: Observe color changes and measure the diameter of any discoloration. Use the supernatant of CV026 bacteria as a negative control.

## Text S4. LC-MS analysis of PCS

Three groups of samples were set up:

a. *Paramecium* secretion (PCS) as described in Text S1,

b. The supernatant of the PAO1 culture incubated in sterile water for 12 hours (MS),

c. The supernatant of PAO1 culture incubated in sterile 50% PCS for 12 hours (PMS).

This setup aims to determine the small molecules accepted by PAO1 in PCS through component analysis and differential comparison. After pretreatment, samples were loaded onto an LC-MS system, with water as phase A and acetonitrile (purity 99.99%) as phase B. The injection temperature was 25°C, and the sample volume was 3 μL. The Thermo Scientific™ Compound Discoverer™ software was used to process the mass spectrometry data, and a Thermo Scientific™ Q Exactive Plus LC-MS system was used for multi-level data acquisition. Comparative analysis was performed using metabolomics, lipidomics, environmental, and natural product databases. Small molecules with differential contents were identified through comprehensive comparison, excluding typical nutrients, unstable compounds, toxic substances, and metabolic waste, and then tentatively identified as cyanuric acid.

## Text S5. Quantification of cyanuric acid

A total of 100 mL of particulate-containing solutions were filtered using aseptic techniques and subsequently subjected to freeze-drying in a freeze-dryer. The remaining solid was dissolved in 2 mL of deionized water through ultrasonication. Cyanuric acid solutions with concentrations ranging from 1 to 500 ng/mL were prepared in deionized water. An Agilent 1100 liquid chromatography system, coupled with a Waters Quattro micro™ Micro Mass triple quadrupole mass spectrometer, was used to examine the samples. Quantification was conducted using internal standard calibration. To establish the presence of cyanuric acid, the ion with a mass-to-charge ratio (m/z) of 42 was chosen as the quantification ion, resulting in a specific ion ratio. Each analyte's response was adjusted to account for matrix effects, extraction losses, and instrument variability. The area response of each sample was multiplied by the ratio of the concentration of the internal standard to the area of the internal standard. The chromatograms were examined using the TargetLynx program (Waters, Milford, MA, USA). The limit of quantification (LOQ) was established as the minimum concentration on the solvent standard curve, which measured 1 ng/mL. The limit of confirmation (LOC) for cyanuric acid was determined to be 1 ng/mL, with a signal-to-noise ratio of at least 5. The limit of detection (LOD) for cyanuric acid was verified to be 1 ng/mL, with a signal-to-noise ratio of at least 6.

Chromatographic conditions: methanol-water = 10:0; Flow rate: 0.3 mL/min; Column temperature: 30°C; Chromatographic column: Agilent Poroshell 120 EC-C18 50 × 4.6 mm, 2.7 μm; Sample volume: 1 μL.

Ion source: electrospray ionization (ESI) source; Negative ion mode, monitoring mode: MRM; Ionization voltage: 2.5 kV; Ion transport tube temperature: 380°C; Heating temperature of auxiliary gas: 380°C; Sheath gas flow rate: 15 Arb; Auxiliary gas flow rate: 50 Arb.

## Text S6: Elastase Assay

The *P. aeruginosa* PAO1 strains were cultured overnight with shaking at 37°C in LB broth[1]. The cultures were diluted at a ratio of 1:100 in 3 mL of LB broth. Subsequently, CA, HSLs, or an equivalent volume of DMSO were added to each culture. The cultures were incubated overnight at 37°C with agitation. A 1 mL aliquot was taken from each culture, and the cells were collected by centrifugation at 16,000 × g. The supernatant was filtered through a 0.22μm membrane. Then, 100 μL of the filtrate was combined with 900 μL of a solution containing 10 mM Na_2_HPO_4_ and 10 mg of elastin-Congo red substrate (Sigma-Aldrich). The mixture was incubated at 37°C for 2 hours, followed by centrifugation at 16,000 × g for 10 minutes. The supernatant was discarded, and the absorbance at 495 nm was measured using a water blank.

## Text S7: Exopolysaccharide Assay

The overnight culture of PAO1 was harvested by centrifugation at 15,000 × g for 5 minutes and then boiled at 100°C for 15 minutes.  Following boiling, 20 µL of Proteinase K was added, and the sample was incubated at room temperature for 1 hour. Next, 100 µL of 85% Trichloroacetic acid (TCA) was added, and the mixture was incubated on ice for 30 minutes before centrifugation at 15,000 × g for 20 minutes. The supernatant was collected, and an equal volume of 95% ethanol was added. This mixture was incubated at 20°C for 1 hour and then centrifuged again at 15,000 × g for 20 minutes. The precipitate was collected, washed twice with 95% ethanol, and centrifuged at 15,000 × g for 20 minutes. The final precipitate was dissolved in 1 mL of deionized distilled water and stored at -20°C. Total exopolysaccharide was estimated using the phenol–sulfuric acid method [2].

## Text S8: QPCR of PAO1

In addition to measuring QS activity, we performed semi-quantitative analysis using the QPCR method to examine the synthesis genes of signal molecules, transcription regulation genes, and quorum control genes in the quorum sensing network of *P. aeruginosa* PAO1. Bacteria were cultured in LB medium containing 100 nM cyanuric acid. After 12 hours of culture, total RNA was extracted using the bacterial total RNA extraction kit (Sangon Biotech, CN). The TransScript All-in-One First-Strand cDNA Synthesis SuperMix for qPCR kit (TransGen Biotech, CN) was used to synthesize cDNA. The cDNA synthesis reaction conditions were as follows: 15 minutes at 40°C for reverse transcription and 30 seconds at 85°C for inactivating the reverse transcriptase. RT-qPCR amplification was performed using the TOP Green qPCR Super Mix kit (TransGen Biotech, CN). The total reaction volume was 20 μL, including 1 μL of cDNA, 10 μL of 2× TransStart Green qPCR SuperMix, 0.4 μL of forward primer (10 μM), 0.4 μL of reverse primer (10 μM), and nuclease-free water. The amplification conditions were as follows: 1 cycle of initial denaturation at 95°C for 10 minutes, 35 cycles at 95°C for 10 seconds, annealing at 57°C for 30 seconds, and a final extension at 72°C for 15 seconds. The housekeeping gene 16S was used as a reference gene[3]. The primer sequences used in this study are shown in Table S2. The relative gene expression was determined using the comparative Ct (2^-ΔΔCt) method.

## Text S9: Construction of *P. aeruginosa* PAO1-ΔLasI/ΔRhlI

Using the PAO1 strain as a template, PCR amplification was carried out with del-RhlI-F/R, del-LasI-F/R, LasI-L-F/R, LasI-R-F/R, RhlI-L-F/R, and RhlI-R-F/R primers, resulting in the fragments RhlI-D and LasI-D. Using three fragments of LasI-L/D/R as templates and the primers LasI-L-F and LasI-R-R, the knockout fragment LasI-LDR was obtained, purified, and concentrated to over 300 ng/μL by PCR amplification and purification (eluted with deionized water). Similarly, using RhlI-L-F and RhlI-R-R as primers, the knockout fragment RhlI-LDR was obtained and purified to a concentration above 300 ng/μL. PAO1<pSim6-GmR competent strains were prepared by electroporation, and the RhlI-LDR or LasI-LDR fragment was introduced by electroporation. Monoclonal clones were cultured in a resistant medium, sequenced, and expanded; plasmids were cultured in a non-resistant medium, resulting in the sequencing-verified strains PAO1-ΔLasI-cmr and PAO1-ΔRhlI-cmr.

## Text S10: Construction of fluorescent plasmid reporter strain

The pACYCDuet-1 plasmid served as the expression vector for the signal molecule receptor proteins LasR and RhlR. The promoter regions of lasB and rhlA, specifically at positions -197 to +348, were introduced upstream of the GFP operon to create a fusion protein. Ribosome binding site sequences were added to enhance the translation of luciferase mRNA. After confirming the plasmids by sequencing, the *E. coli* BL21 (DE3) competent cells were co-transformed with the plasmids. This experiment utilized LB agar supplemented with chloramphenicol at a concentration of 34 μg/mL. Durable colonies were selected for propagation and storage. The strain was incubated overnight at 37°C and subsequently grown in LB medium with a 1% inoculum until the optical density at 600 nm (OD600) reached 0.8. The culture was then incubated at 16°C for 24 hours following the addition of 0.1 mg/L IPTG and the appropriate signal molecules. Subsequently, 200 μL of the bacterial culture was introduced into each well of a 96-well microplate. The OD600 and fluorescence intensity at an emission wavelength of 507 nm, with an excitation wavelength of 395 nm, were measured using a multifunctional microplate reader (Varioskan LUX, Thermo Fisher Scientific, USA).

## Text S11: Molecular docking

Through experiments, we speculated that cyanuric acid likely binds to the LasR signal molecule receptor of *P. aeruginosa*, thereby activating downstream receptors such as LasB and LasI. To verify this hypothesis, we used molecular docking software to simulate the docking of cyanuric acid with LasR. Due to the central symmetry of the cyanuric acid molecule, we also simulated the docking of other typical quorum sensing signal receptors with it. Molecular docking simulations were performed using AutoDock v.4.2 to investigate ligand-receptor interactions at the binding sites of LasR, RhlR, TraR, and CviR. The 3D structures of the co-crystal complexes with their cognate ligands 3OC12-HSL, C4-HSL, and C6-HSL were obtained from the Protein Data Bank (PDB codes: 3IX3, 8B4A, 1H0M, 3QP1). The PDB formatted complexes were visualized and prepared using the structure preparation tool in the Sybyl X 2.0 software package. The cognate ligands and all water molecules were removed from the complexes. The pH was set to 7, hydrogen atoms were added, and side chain amides and imidazoles were protonated. The Amber force field was used to assign partial atomic charges to the protein. The geometric structure of the cognate ligand (3OC12-HSL) was optimized using the conjugate gradient minimization algorithm with Gasteiger charges in Sybyl X 2.0. The Lamarckian genetic algorithm (LGA) was used for ligand conformational search to obtain docking calculation parameters. The Autogrid4 program (included in AutoDock) was used to calculate the potential docking grid, and the bpdqt file representing the docking pose with the highest binding energy was selected. Ligand hydrogen bonding, 2D ligand-protein interactions, and 3D docking poses of each docking complex were analyzed and visualized using Pymol V.2.5.4, Discovery Studio Client V.19.0, and Molecular Operating Environment V.2022, respectively.

## Text S12: Molecular dynamics simulation

The GROMACS 2022.4.1 program was used to simulate the docking receptor-ligand complex. The stability of receptor molecules and receptor-ligand complexes was investigated using molecular dynamics (MD) simulation. The Charmm36-jul2022 force field was employed to construct the topology for proteins, whereas the sobtop tool was utilized to generate the topology for ligands[4]. The protein-ligand entity was solvated in the center of the box using SPC/E (simple point charge/expansion) water molecules. Counter ions were added to neutralize the entire system. The steepest descent minimization approach was employed to minimize the simulation system, consisting of both the solvent and solute, over 500,000 steps, followed by an equilibrium state of 100 ns in the NPT and NVT conservation ensembles. Once the system reached equilibrium at a temperature of 300K and a pressure of 1 bar, it was run for 500,000 ps. The acquired trajectory was further examined to compute the root mean square deviation (RMSD), root mean square fluctuation (RMSF), hydrogen bonds, Lennard-Jones potential (LJ-SR), distance of residues, and radius of gyration (Rg). The binding energy of the ligand complex was determined using the g_mmpbsa tool in GROMACS, based on the trajectory generated by MD simulation, using the molecular mechanics Poisson-Boltzmann surface area (MM/PBSA) method.

## Text S13: Anammox bioreactor

A set of four sequencing batch reactors (SBRs) with a uniform volume of 1 L each were used as vessels for cultivating anammox sludge. The experiment spanned a total of 60 days, divided into four distinct phases. Throughout the experiment, a consistent temperature of 35°C was maintained using a water bath. Cyanuric acid was added to the three experimental reactors at final concentrations of 50 nM, 500 nM, and 5 mM, respectively. The first reactor (R1) served as the control. The inoculum sludge consisted of 10% finely dispersed mature anammox granular sludge and 90% anaerobic sludge from a secondary sedimentation tank aged for six months (obtained from Liede Wastewater Treatment Plant in Guangzhou). The initial mixed liquor suspended solids (MLSS) and mixed liquor volatile suspended solids (MLVSS) concentrations of the inoculum sludge were 17.78 mg/mL and 6.04 mg/mL, respectively.

To replicate the composition of nitrogenous wastewater in the reactors, the concentrations of NH_4_^+^-N and NO_2_^-^-N were set at 240 mg/L and 220 mg/L, respectively[5] (Table S3). To optimize the load capacity when the concentration of NO_2_^-^-N in the effluent falls below 20 mg/L, the hydraulic retention time (HRT) was adjusted accordingly. The experiment was divided into four phases: 12 hours (1-14 days, phase I), 8 hours (15-29 days, phase II), 6 hours (30-44 days, phase III), and 4.8 hours (45-60 days, phase IV). Samples were collected daily from the effluent to quantify the concentrations of NH_4_^+^-N, NO_2_^-^-N, NO_3_^-^-N, and pH levels. After each phase, a 15 mL sample from the homogenized sludge-water mixture was collected to analyze particle size, extracellular polymeric substances (EPS), MLSS, and MLVSS. To ensure repeatability, an additional set of four identical reactors was used simultaneously, following the same methods.

## Text S14: Chemical analysis method

The analysis of NH_4_^+^-N, NO_2_^-^-N, and NO_3_^-^-N were performed using spectrophotometric methods according to the Chinese National Environmental Protection Standards (HJ535-2009, GB 7493-87, HJ/T 346-2007). The determination of MLSS and MLVSS followed the Standard Methods for the Examination of Water and Wastewater, 22nd Edition. The pH value was determined using a pH meter (PB-10, Sartorius, Germany). The process of extracting EPS from the sludge-water mixture was carried out utilizing a modified heat extraction technique[6]. The protein concentration in EPS (PN) was quantified using bovine serum albumin as the reference material and employing the Lowry technique[7]. The protein concentration in EPS (PN) was quantified using bovine serum albumin as the reference material and employing the Lowry technique[8]. The analyses mentioned above were conducted in triplicate, and the resulting average results were documented. The distribution of organic matter in the EPS extraction solution was observed using excitation-emission matrix (EEM) fluorescence spectroscopy (F-7000, Hitachi, Japan) after a dilution of 16 times. The emission spectra were obtained using scanning with a step size of 10 nm, ranging from 200 to 500 nm. Similarly, the excitation wavelength was adjusted incrementally by 0.5 nm, also ranging from 200 to 500 nm. The excitation and emission slits were maintained at 5 nm for all measurements. The scanning speed for all measurements was set to 1200 nm/min. The regions influenced by Rayleigh and Raman scatter of both first and second order were eliminated using interpolation as described in Bahram *et* al.'s study[9]. Subsequently, the fluorescence regional integration (FRI) technique was used to analyze the EEM dataset, which was divided into five distinct excitation-emission zones as per the outcomes reported by Chen et al. MATLAB 2022.a (Mathworks, Natick, MA) was utilized for visualizing the fluorescence spectra[10].

The sludge's particle size after each phase was assessed using a laser diffraction particle size analyzer (Mastersizer 3000, Malvern Panalytical, UK) within the 0.02-2000 μm range. The median particle size D(0.5) was used as a key indicator.

SAA (specific activity of anammox) analyze was conducted in 50 mL serum bottles. Biomass was gathered from the four SBRs and isopycnic transferred in culture solution with NH_4_^+^-N and NO_2_^-^-N at 100 mg-N/L and different CA correspond. The content changes of NH_4_^+^-N, NO_2_^-^-N, and NO_3_^-^-N were determined, and SAA was calculated by nitrogen removal rate. The tested biological samples were returned to SBRs.

## Text S15: qPCR of AnAOB genes

*Candidatus Brocadia* was identified as the primary anammox species via diversity analysis. A set of genes associated with denitrification capability within the microbiota of anammox systems, namely *hzsB, narG, napA, nirS, nirK,* and *nosZ,* were chosen for analysis. The gene copy counts of these selected genes were amplified and quantified using the primers specified in Table S2[11–15]. The relative quantification of genes was performed using a fluorescence quantitative PCR instrument with the SYBR Green dye method.

## Text S16: Community diversity of anammox

Seed sludge samples and fresh sludge were collected after the conclusion of the fourth phase specifically for high-throughput 16S rRNA gene amplicon sequencing. The amplification of the V3-V4 region of bacterial 16S rRNA was conducted using the primers 338F (5′-ACTCCTACGGGAGGCAGCA-3′) and 806R (5′-GGACTACHVGGGTWTCTAAT-3′). The polymerase chain reaction (PCR) products were subjected to electrophoresis analysis using a 2% agarose gel. The PCR products were quantified using the QuantiFluor™-ST Blue Fluorescence Quantitation System (Promega) after preliminary quantification from electrophoresis. The quantified products were then combined in quantities suitable for the sequencing needs of each sample. Efficient sequences were carefully selected, followed by normalization and grouping of information from each sample into operational taxonomic units (OTUs) using a 97% similarity criterion for the trimmed sequences. Diversity analysis was conducted using the online bioinformatics analysis tool Majorbio Cloud (www.i-sanger.com).

## Text S17: HSL quantification in AnAOB

The analysis of HSL in the sludge complex was conducted on the 45th day of the experiment. A 20 mL sample of sludge was freeze-dried for 36 hours. The dried sample was resuspended in 20 mL of ethyl acetate using ultrasonic waves to extract HSLs from the biomass. The mixture was centrifuged at 4,000 rpm for 15 minutes, and the supernatant was collected. This extraction process was repeated three times. The collected ethyl acetate extracts were then subjected to spin-evaporation at 35°C. The residue was dissolved in a centrifuge tube using chromatographic-grade methanol and subsequently dried under nitrogen gas, followed by the addition of 1 mL of methanol[16]^,22^.

The extracted materials underwent analysis using a liquid chromatography-tandem mass spectrometry (LC-MS/MS) system, specifically the SCIEX QTRAP 4500 instrument. The separation of the samples was performed using a BEH C18 column with dimensions of 1.7 μm and 2.1×50 mm, operating at a flow rate of 0.2 mL/min. The mobile phase consisted of two solvents: solvent A, containing 2 mM ammonium formate and 0.1% formic acid, and solvent B, consisting of methanol with 0.1% formic acid. The eluate was ionized in positive ion mode using electrospray ionization (ESI) (Table S4-5). The accurate quantification of the HSLs was achieved by measuring the intensity of the collision-activated product ions[17].

## Text S18: Metagenome of anammox under high pressure start-up

Following the high pressure start-up process of anammox, comprehensive metagenomic characterization was performed to elucidate microbial community dynamics and quantify the abundance of functional genes involved in nitrogen metabolism. The experimental procedures adhered to Illumina’s standard protocol, encompassing sample quality assessment, library construction, quality validation, and high-throughput sequencing. Qualified genomic DNA was first fragmented, followed by end repair, 3′-end adenylation, adaptor ligation, product purification, and fragment selection. After amplification and a second round of purification, sequencing libraries were constructed and subjected to quality control before being sequenced on the Illumina platform.

Raw sequencing reads underwent quality filtering and control to generate clean reads, which formed the foundation for subsequent bioinformatic analysis. Clean reads were assembled into contigs using MEGAHIT, with sequences shorter than 300 bp discarded. Assembly quality was evaluated using QUAST. Coding regions were predicted using MetaGeneMark (<http://exon.gatech.edu/meta_gmhmmp.cgi>, Version 3.26) with default parameters, and a non-redundant gene catalog was generated using MMseqs2 (<https://github.com/soedinglab/mmseqs2>, Version 12-113e3), applying a protein sequence identity threshold of 90% and a coverage threshold of 80%.

Functional annotation was conducted using both general-purpose and specialized databases, while taxonomic analysis provided detailed insights into species composition and relative abundance. Final analysis and reporting were conducted via the BMKCloud platform (<http://www.biocloud.net/>).

## Text S19: MD analyses of C4-HSL and CA induce RhlR

CA was found to energetically form hydrogen bonds with TRP68 and ASP81, whereas C4-HSL interacted with TYR64 and ASP81 via hydrogen bonding (Fig. S6a-b). In both complexes, RhlR-CA and RhlR-HSL, the distances between ASP81 and TYR64, as well as TRP68, showed similar changes, indicating that ligand binding enhances receptor stability (Fig. S5d, 5f). Structural analysis based on RMSD indicated that the structure of RhlR exhibited greater flexibility than LasR (Fig. S8e). Both complexes demonstrated a reduced radius of gyration during simulation, indicating a shift towards a more compact structure compared to unbound RhlR (Fig. S6d). Moreover, The RMSF of residues in the RhlR-CA complex exhibited minimal fluctuation, indicating the structural stability of receptors (Fig. S6c). MMPBSA analysis yielded ΔGtotal values of -21.49 kcal/mol for RhlR-HSL and -16.59 kcal/mol for RhlR-CA. The ΔGgas values were -30.49 and -21.32 kcal/mol, while the values for ΔGsolv were 9.00 and 4.73 kcal/mol, implying that the holistic binding strength of RhlR-CA was comparable to that of RhlR-HSL (Fig. S6e). PHE101, TRP108, LEU107, PRO82, and TRP96 exhibited enhanced van der Waals interactions with both ligands. Notably, strong van der Waals forces were observed between TYR72 and TRP68 with HSL in the RhlR-HSL complex. In the RhlR-HSL complex, TYR72, TRP96, and TRP68, along with ASP81 in the RhlR-CA complex, contributed to solvent aversion (Fig. S6f-g). FEL analysis identified a distinct low-energy region for RhlR-CA within a wider low-energy zone for RhlR-HSL (Fig. S6h). This finding indicated that CA restricts RhlR to a narrower range of conformations, most of which overlap with MECs of RhlR-HSL. In contrast, the unbound protein exhibited multiple MECs, which rarely overlap with that of RhlR-HSL.

## Figure S1: The HSL-mediated group sensing pathway in *Pseudomonas aeruginosa*.


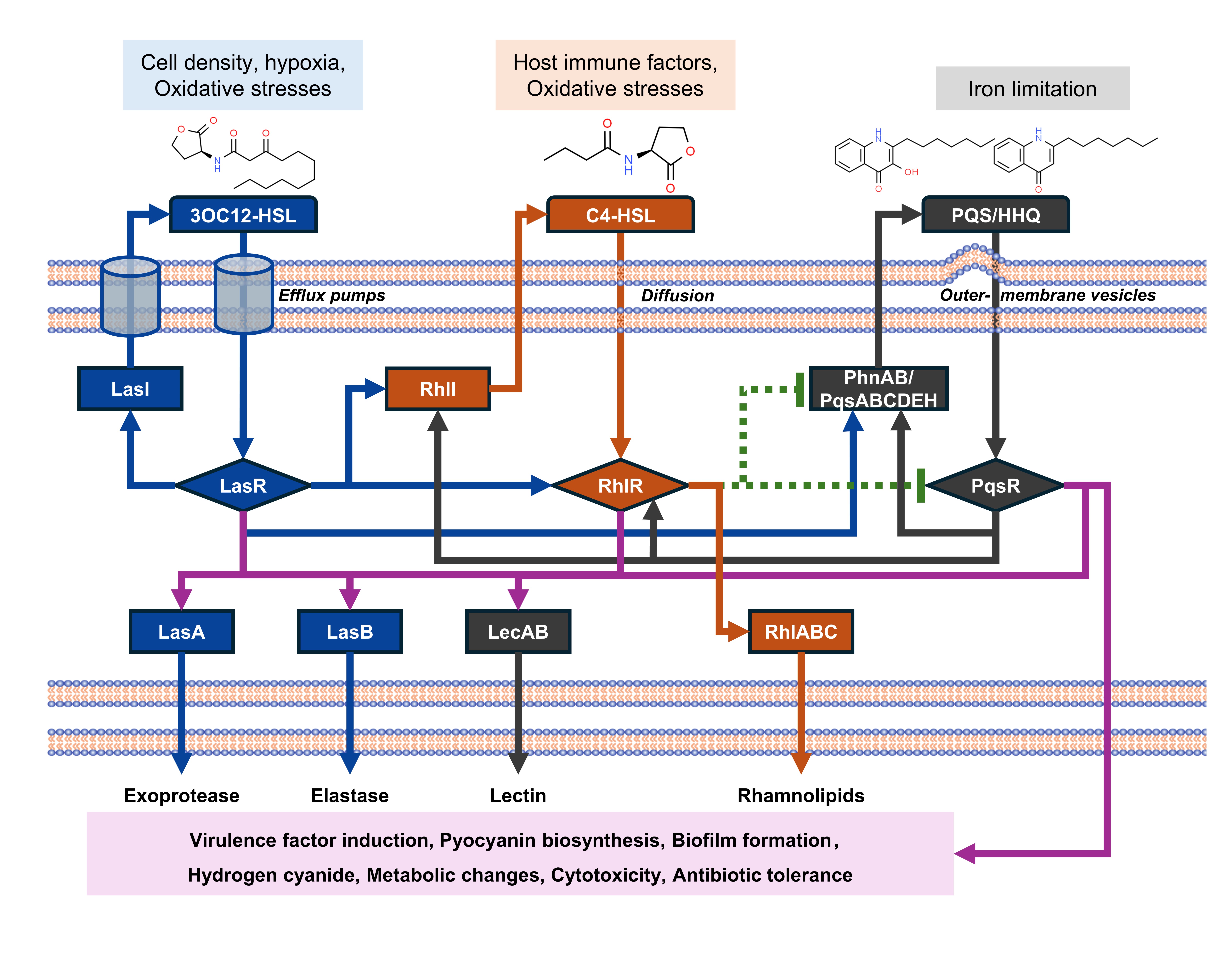


## Figure S2: Analysis of PCS and impact of CA on PAO1 growth


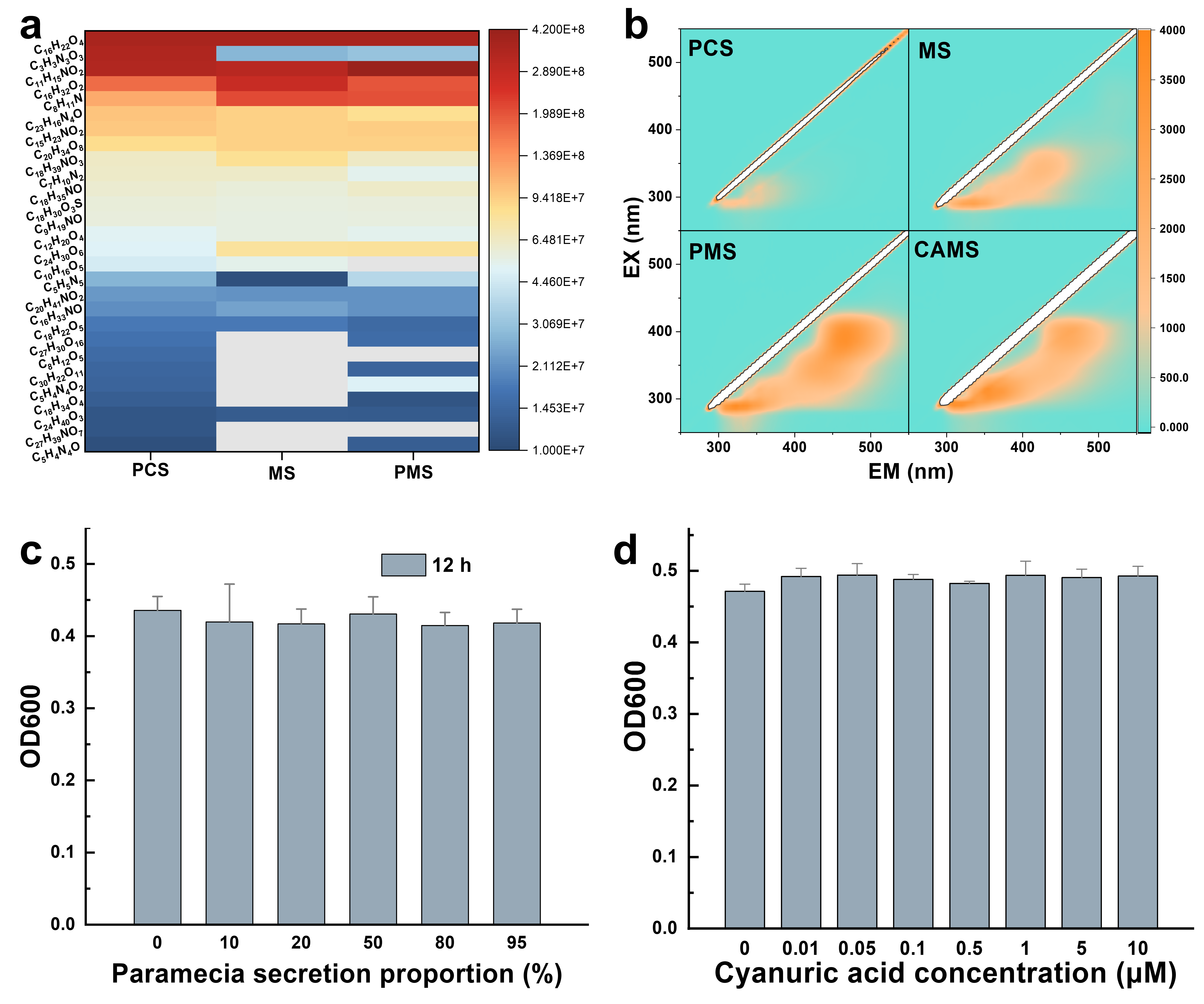


**a**, Comparison of PSC solution and PAO1 secretion in PBS (MS) and PBS containing 50%PCS (PMS) through LC-MS analysis. **b**, Determination of extracellular secretion of PCS, PAO1 in PBS and PBS containing 50%PCS and 100nM cyanuric acid (CAMS) through excitation-emission-matrix spectrum (EEM). **c**, The effect of PCS in PBS on PAO1 cell density. **d**, The effect of CA in 1% LB culture on PAO1 cell density and total hydrolysate proteinase.

## Figure S3: Source and degradation of CA





Cyanuric acid in the environment primarily originates from the stepwise hydrolysis of melamine derivatives (via intermediates like ammeline and ammelide), degradation of herbicides such as atrazine (which forms hydroxyatrazine and reacts further with ammonium chloride or methyl compounds), and abiotic hydrolysis of trichloroisocyanuric acid with water to yield isocyanic acid. Its degradation involves microbial enzymes (e.g., AtzD, AtzE, AtzF) that cleave the triazine ring, ultimately breaking it down into inorganic compounds like ammonium ions and carbon dioxide, which re-enter natural biogeochemical cycles.

## Figure S4: CA affects the QS gene expression in PAO1


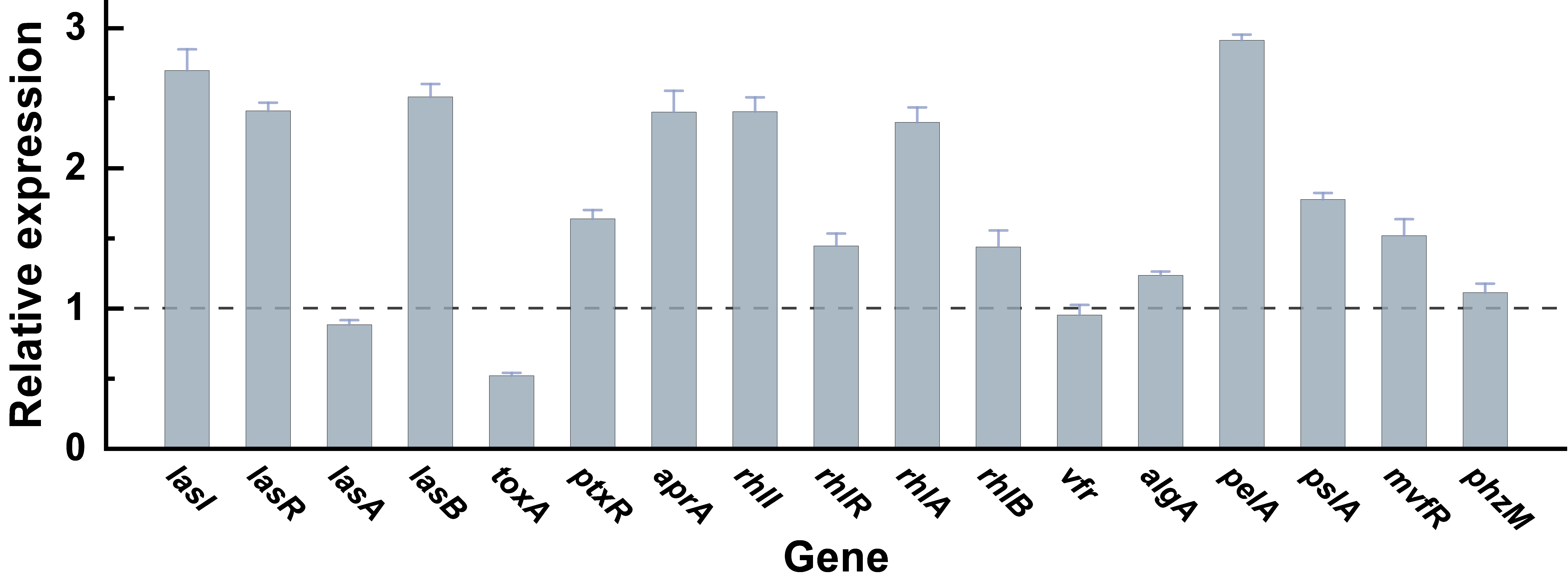


The effect of 100nM cyanuric acid on the gene expression level associated with quorum sensing.

## Figure S4: Molecular docking and bonding


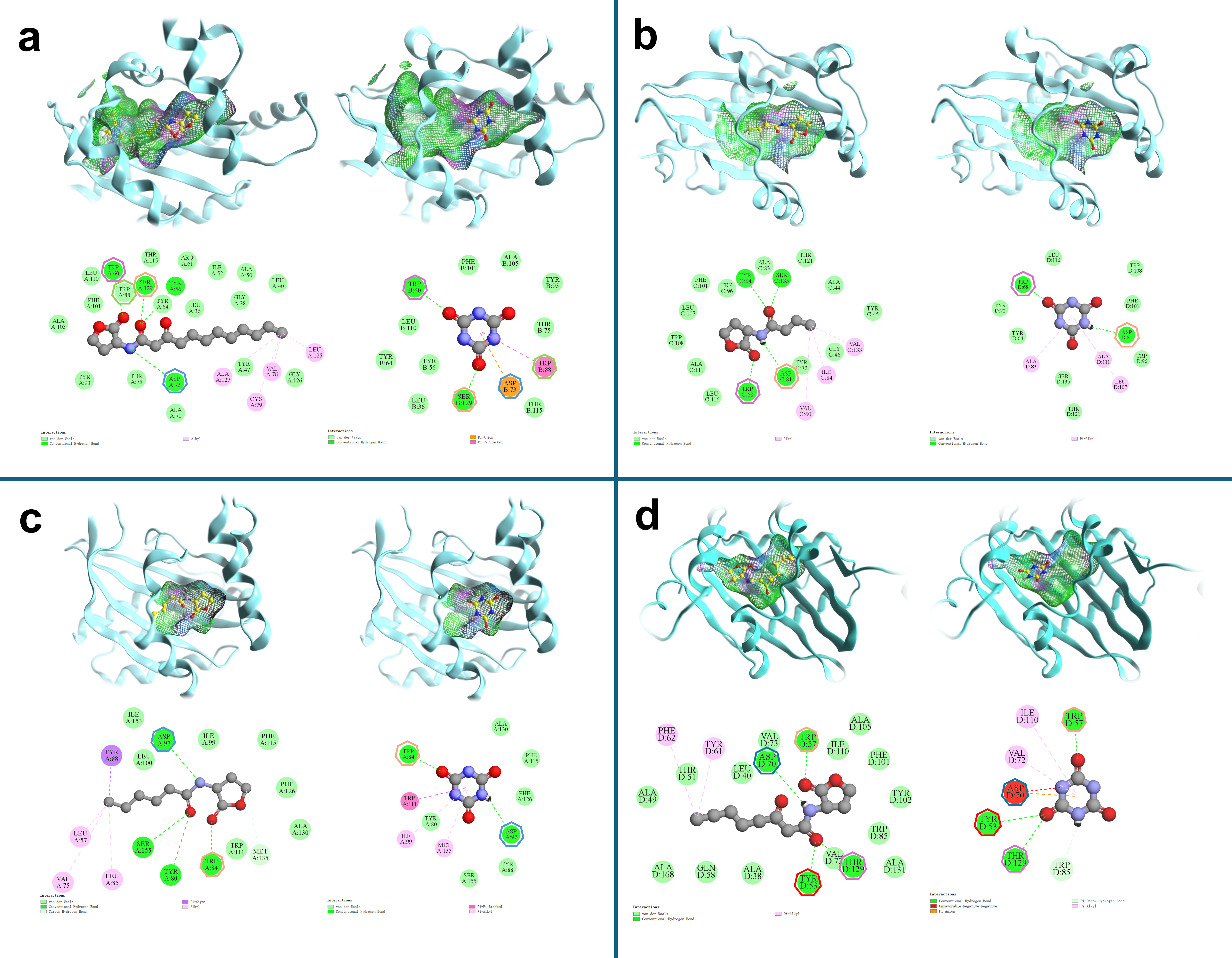


**a-d**, The interaction and attachment of proteins (LasR, RhlR, CviR, TraR) to ligands (HSL and cyanuric acid).

In the binding of LasR and RhlR with HSL ligands, the primary strong binding occurs in the lactone ring and its adjacent carbonyl group, and the acyl tail end is accompanied by weak hydrophobic interaction. CA can build similar bond with receptor imitating as the lactone ring of HSL. However, TYR88 of CviR formed a solid π-sigma at the tail carbon of the acyl group, which may be the reason why KYC55 can only detect longer HSLs. Obviously, CA can't establish a similar connection. In the simulation of TraR, ASP70 forms hydrogen bonds with HSL but has strong electrostatic repulsion with CA. As a result, CA can't be stably bound in the pocket, and it can't mediate TraR activity.

## Figure S6: The distance between important pairs of residues and LJ-SR of receptors.


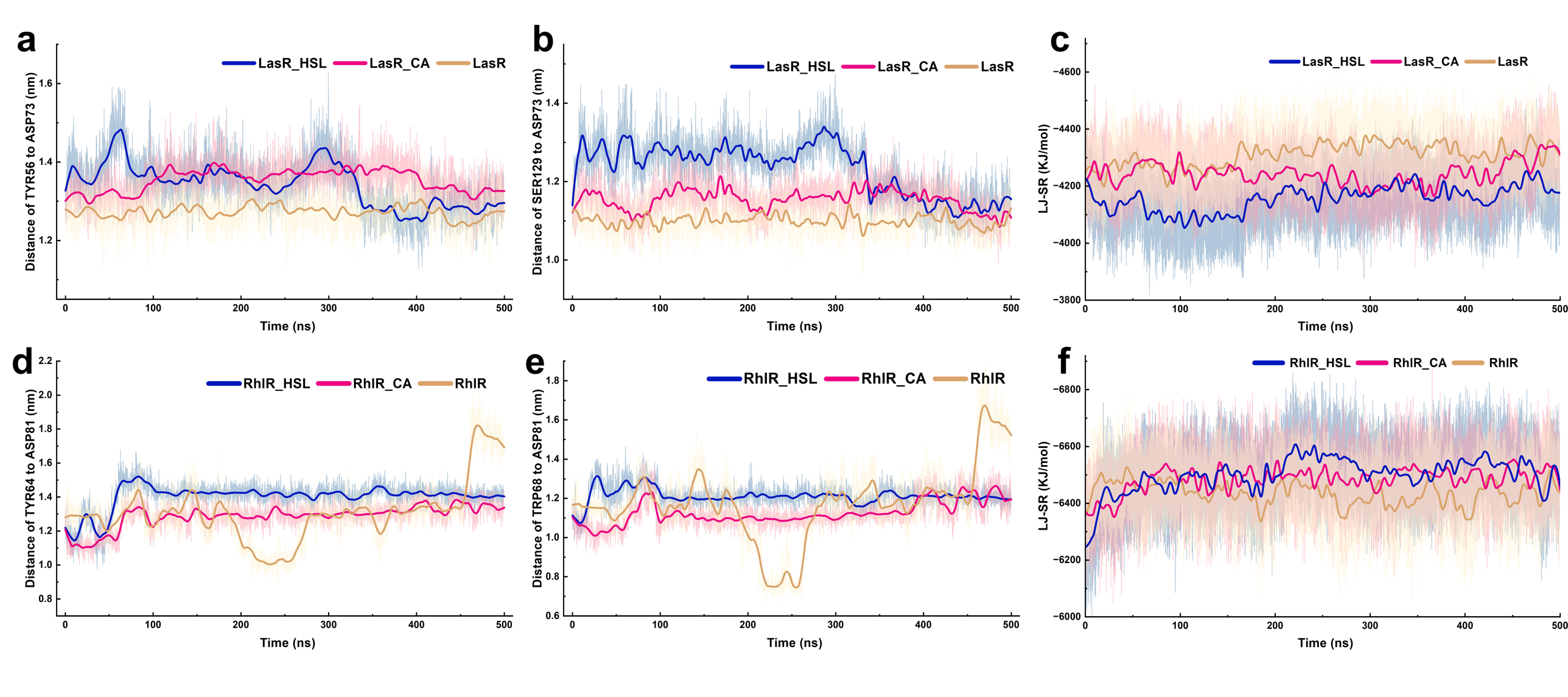


**a,b,d,e**, The change in distance between important pairs of residues during the 500ns MD simulation. **c,f,** LJ-SR of LasR/RhlR and its ligand complexes observed during the MD simulation.

## Figure S7: MD simulation analysis of RhlR





**a-b**, The hydrogen bond between LasR and the C4-HSL and cyanuric acid. **c,** RMSF of LasR and its ligand complexes observed during the MD simulation. **d,** The protein gyration radius changes during the receptor and complex simulation**. e,** The binding energy in calculating MMPBSA of the complex. **h,** Alterations in the free energy landscape of RhlR and its ligand complexes observed during the MD simulation.

## Figure S8: Hydrogen bonding and MD simulation analysis of CviR and TraR


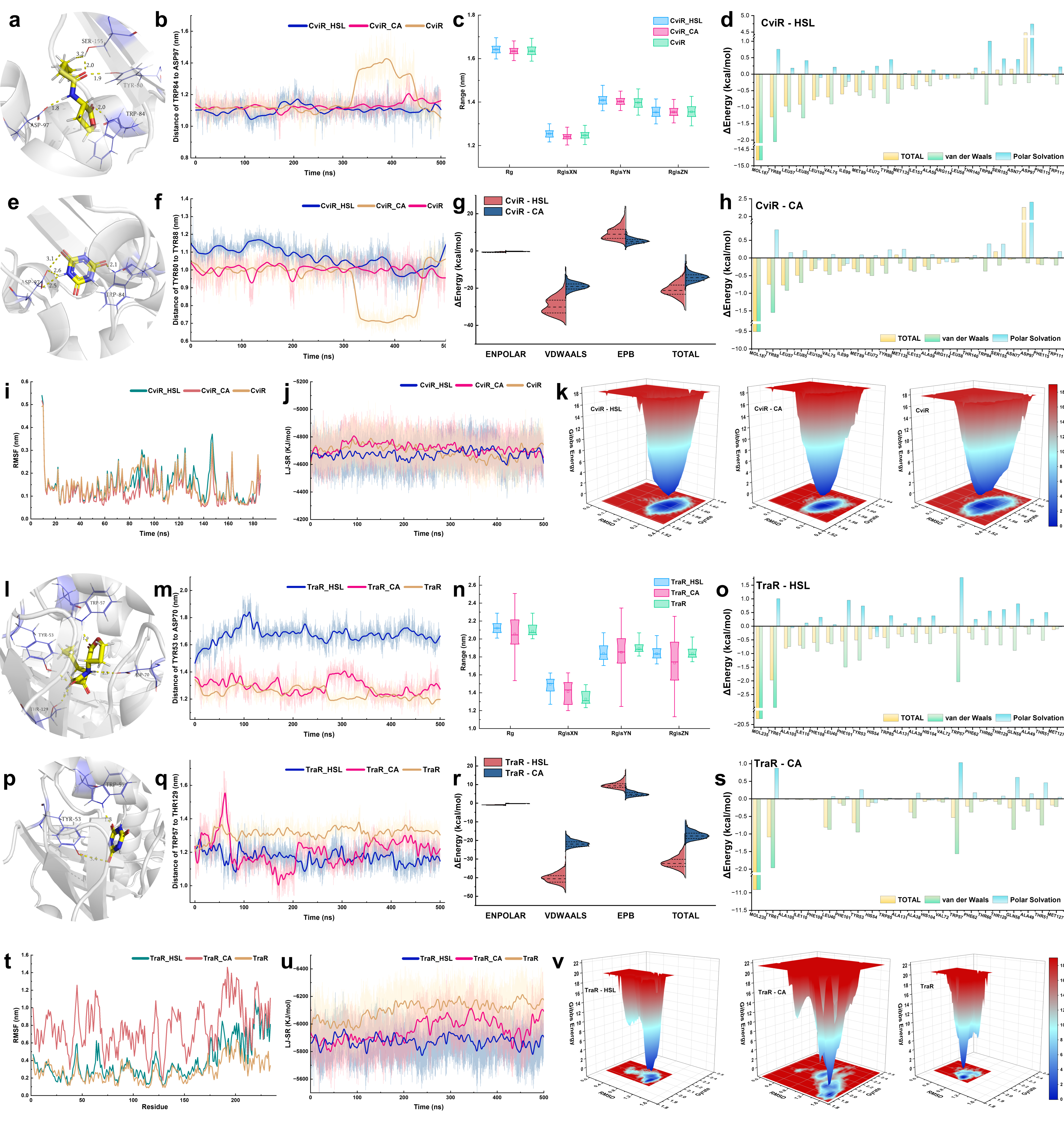


**a-b**. The hydrogen bond between CviR/TraR and the natural autoinducer (3-oxo-C6-HSL) and cyanuric acid, the change in distance between important pairs of residues during the 500ns MD simulation, the contribution of residues in the complex to the binding energy, the distribution of binding energy in the calculation of complex MMPBSA, and the change in protein gyration radius during the simulation of the receptor and complex. **c-d**. Alterations in the free energy landscape, repetitive energy (LJ-SR), and root mean square fluctuation (RMSF) of CviR/TraR and its ligand complexes were observed during the MD simulation.

Although both HSL and CA are combined with CviR in the pocket, their structures are obviously finer. In the steady state, there is no noticeable difference in the critical residue distance between the three amino acid. The overall change of CviR-HSL in protein gyration radius is close to that of empty protein. From the RMSF results, it can be seen that the introduction of CA in TraR caused significant fluctuations in the structure, which is impossible for achieving a stable combination.

## Figure S9: Molecular dynamics simulation analysis

**

**

**a-d**, The RMSD, RDF, SASA and RG of proteins (LasR, RhlR, CviR, TraR) to ligands (HSL and cyanuric acid).

## Figure S10: 3D-EEM of extracellular secretion of AnAOB


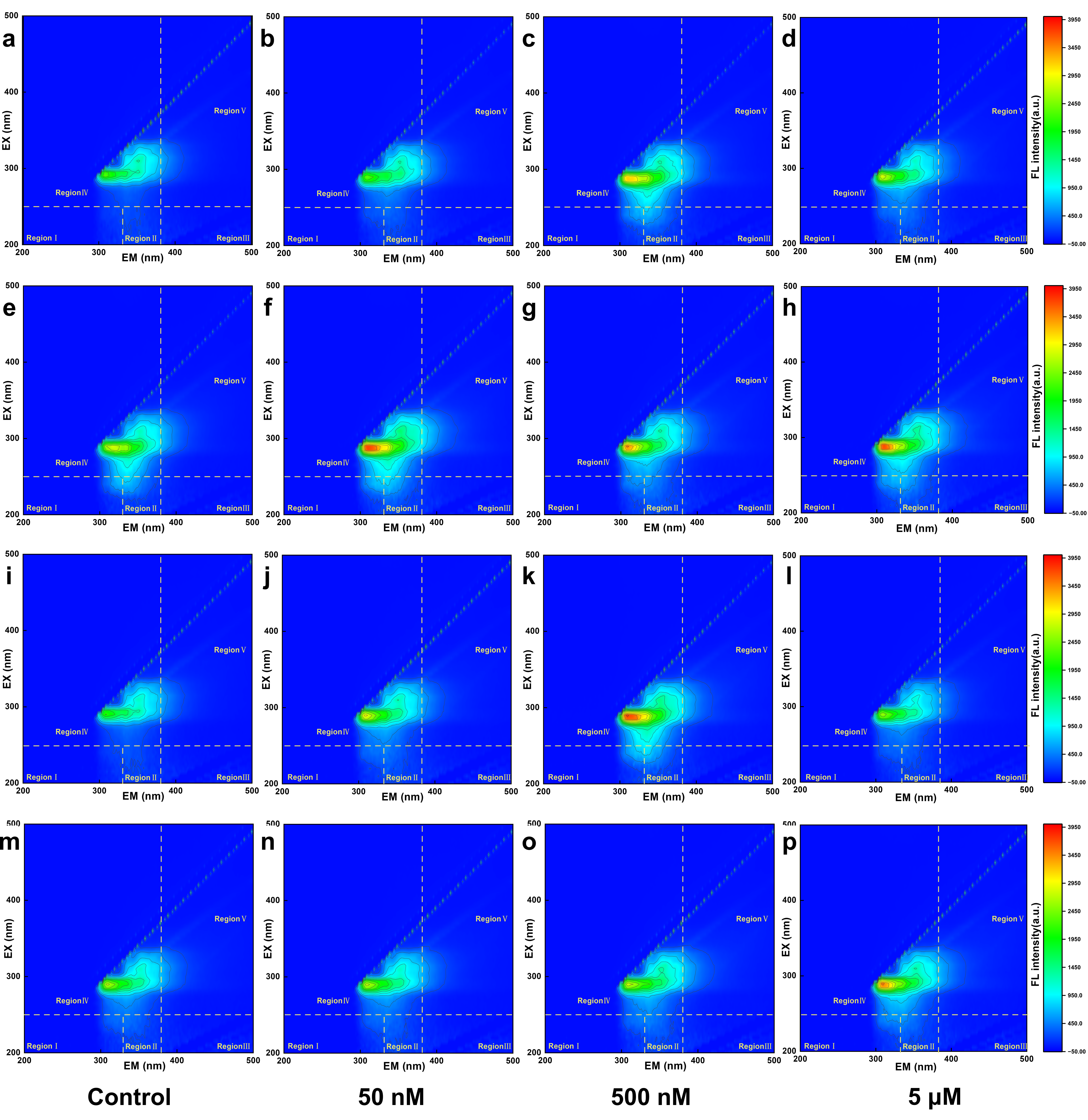


EEM fluorescence spectroscopy characteristics of extracellular secretion of sludge at different stages.

## Figure S11: Alteration of pH, MLSS and MLVSS





**a-c,** The effect of cyanuric acid on pH, MLSS and MLVSS at the end of each phase

## Figure S12: Species diversity analysis of AnAOB


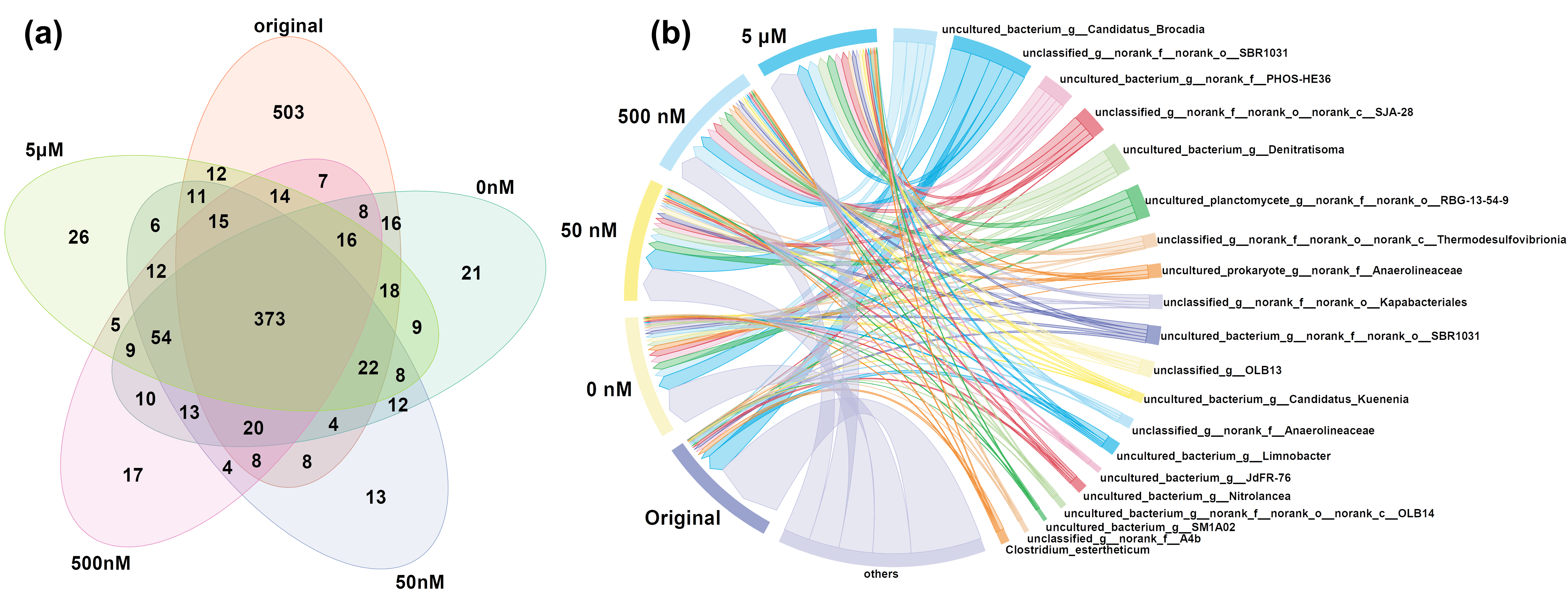


**a**, Distribution of common species in each sample**. b**, Distribution of microorganisms at the species level.

## Figure S13: Quick Start-up of Anammox under High Pressure





a, The concentration of inorganic nitrogen in the effluent and the reactor's NRR. b-c, The distribution of microorganisms in the seed sludge and post-process sludge at the phylum and species levels.

## Table S1: Mass spectrometry parameters of cyanuric acid quantification

| Compound | Retention time (min) | Scanning mode | parent ion(m/z) | daughter ion (m/z) | RF(V) | CE(V) |  |
| --- | --- | --- | --- | --- | --- | --- | --- |
|  |
| Cyanuric acid | 1.13 | ESI^-^ | 127.9 | 41.9^*^/84.9 | 67 | 12.92/10.23 |  |

## Table S2: Primer used in this study

| Primer name | primer sequences 5’→3’ |
| --- | --- |
| *P. aeruginosa*-16S-F1 | GCGTGGAAAAGAGCTTCTGG |
| *P. aeruginosa*-16S-R1 | ATTTCCGGCAGCACCGTATC |
| LasI-F | TGTTCAAGGAGCGCAAAGG |
| LasI-R | ATGGCGAAACGGCTGAGTT |
| LasR-F | AGCGACCTTGGATTCTCGAAG |
| LasR-R | CGAAGAACTCGTGCTGCTTTC |
| LasA-F | TGCATTTCTCGCTGCTCTAC |
| LasA-R | GCGACAGTCGTTGTCGTAGT |
| LasB-F | AGTTTGGACACGTCGATCAG |
| LasB-R | GCTTGACCTGTTGTTCGTTG |
| toxA-F | GGAGCGCAACTATCCCACT |
| toxA-R | TGGTAGCCGACGAACACATA |
| ptxR-F1 | TTGGGAAACTGCCGGATGAA |
| ptxR-R1 | GTCGCATGACCCTTACCGAT |
| aprA-F | GCTTCAGCCAGAACCAGAAGAT |
| aprA-R | TCGACACATTGCCCTTCAAC |
| rhlI-F | TGCTCTCTGAATCGCTGGAA |
| rhlI-R | GTTTGCGGATGGTCGAACTG |
| rhlR-F1 | GCTGGAGATGTTCTGCTGGT |
| rhlR-R1 | GAAATGGTGGTCTGGAGCGA |
| rhlA-F1 | TTTCCACCTCGTCGTCCTTG |
| rhlA-R1 | CCTGGCCGAACATTTCAACG |
| rhlB -F | CATTTCCTCGACCTGGAGTC |
| rhlB -R | ATCGAGAAAGCGTTGCAGTT |
| vfr-F | GGTTCGGTCACCATCCTCAT |
| vfr-R | GTTCCTGTTCGCTGCCTTC |
| algA-F | AGAACTGAAGAAGCACGACG |
| algA-R | TTCTCCATCACCGCGTAGT |
| pelA-F | GGAACAGCCAGGTAATGGAC |
| pelA-R | TCCAGGGTATCGAGGAACAG |
| pslA-F | CGGTCAGCGAATACAGCTC |
| pslA-R | TTGATCTTGTGCAGGGTGTC |
| MvfR-F | AAACTTCGACGACATGCTGC |
| MvfR-R | TCGTAGAGTTCGCTGAGGAC |
| phzM-F | GACATGGTGCTGTTCTAC |
| phzM-R | TCAGGTAGCTGTAGAAGTC |
| AnAOB-16S-341F  AnAOB-16S -512R | CCTACGGGAGGCAGCAG  TTACCGCGGCTGCTGGCAC |
| Amx_368F  Amx_820R | TTCGCAATGCCCGAAAGG  AAAACCCCTCTACTTAGTGCCC |
| hzsB_396F  hzsB_742R | ARGGHTGGGGHAGYTGGAAG  GTYCCHACRTCATGVGTCTG |
| narG1960m2f  narG2050m2r | TAYGTSGGGCAGGARAAACTG CGTAGAAGAAGCTGGTGCTGTT |
| napA3F  napA3R | CCCAATGCTCGCCACTG  CATGTTKGAGCCCCACAG |
| nirSnF  nirSnr | TACCACCCCGAGCCGCGCGT  GCCGCCGTCRTGVAGGAA |
| nirK876  nirK1040 | ATYGGCGGVCAYGGCGA  GCCTCGATCAGRTTRTGGTT |
| nosZ2F  nosZ2R | CGCRACGGCAASAAGGTSMSSGT  CAKRTGCAKSGCRTGGCAGAA |
| del-RhlI-F | GCGCGAAACGGCTGACGACCTCACACCGCCATCGACAGCGGTACGCCCTGCTCGAGGACTAGTGAACCTCTTCGAG |
| del-RhlI-R | CTCGGCCGGCACGACACGGGGACTTGGTCATGATCGAATTGCTCTCTGAATCCGAGCCGATCATATTCAATAACCCT |
| del-LasI-F | GCATAAATTCTTCAGCTTCCTATTTGGAGGAAGTGAAGATGATCGTACAACGAGCCGATCATATTCAATAACCCT |
| del-LasI-R | ACGCGCCGGCGCGAGCCGACAGGTCCCCGTCATGAAACCGCCAGTCGCTGCTCGAGGACTAGTGAACCTCTTCGA |
| LasI-L-F | CATGGAGTCGGTCCTGCCGA |
| LasI-L-R | CTTCACTTCCTCCAAATAGGAAGCTGAA |
| LasI-R-F | CGGGGACCTGTCGGCTCGCGCCGGCGCGTTCTCTGTCGGAGAGGGA |
| LasI-R-R | GCGCTTCCTGCCCTGGATA |
| RhlI-L-F | GGGTTGACGATGGCCGTCA |
| RhlI-L-R | GTCAGCCGTTTCGCGCACTTT |
| RhlI-R-F | GTGTCGTGCCGGCCGAGAAA |
| RhlI-R-R | GCCGTGGATCCGGCGATCCT |

## Table S3: The formula of inlet water

| Element | Concentration （mg/L） |
| --- | --- |
| NH_4_^+^-N | 240 |
| NO_2_^-^-N | 200 |
| NH_4_CL | 917.14 |
| NaNO_2_ | 985.71 |
| KH_2_PO_4_ | 30 |
| CaCl·2H_2_O | 150 |
| MgSO_4_·7H_2_O | 300 |
| KHCO_3_ | 1250 |
| FeSO_4_·7H_2_O | 11.431 |
| EDTA | 6.25 |
| 2000x Trace element solution | 0.5mL |
| Trace composition of 2000x trace element solution | |
| ZnSO_4_·7H_2_O | 860 |
| CuSO_4_·5H_2_O | 500 |
| NiCl_2_·6H_2_O | 380 |
| H_3_BO_4_ | 28 |
| CoCl_2_·6H_2_O | 480 |
| MnCl_2_·4H_2_O | 1980 |
| NaMoO_4_·10H_2_O | 440 |
| Na_2_SeO_3_·5H_2_O | 300 |
| NaWO_4_·2H_2_O | 100 |

## Table S4: The basic properties of HSLs.

| **Signal type** | **Molecular Structure** | ***m*/*z*** | **LogS** | **LogP** |
| --- | --- | --- | --- | --- |
| C4-HSL | 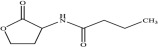 | 172.2 | −1.25 | 0.86 |
| 3OC6-HSL | 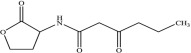 | 214.2 | −1.48 | 0.94 |
| C8-HSL | 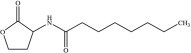 | 228.2 | −2.90 | 3.08 |
| 3OC8-HSL | 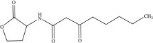 | 242.2 | −2.31 | 2.04 |
| 3OC12-HSL | 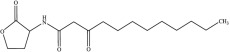 | 298.3 | −3.97 | 4.25 |

## Table S5: Parameters for the procedure of gradient elution.

| **Time (min)** | **Solvent A (%)** | **Solvent B (%)** |
| --- | --- | --- |
| 0 | 50 | 50 |
| 2 | 60 | 40 |
| 4 | 100 | 0 |
| 5 | 100 | 0 |
| 5.8 | 50 | 50 |
| 6.2 | 50 | 50 |

## Table S6: The concentration of CA in water sample

| Item | Rotifer culture | Wastewater | Pearl river | Tap water | Eff of the control | Eff of the treated |  |
| --- | --- | --- | --- | --- | --- | --- | --- |
|  |
| Concentration (ng/L) | N/F | 56±14 | 2870 ± 790 | 265.9± 81 | N/F | 972.16  ±47 |  |

**References:**

1. McCready A, Paczkowski J, Henke B, Bassler B. Structural determinants driving homoserine lactone ligand selection in the *Pseudomonas aeruginosa* LasR quorum-sensing receptor. *Proc Natl Acad Sci U S A* 2019;**116**:245–254. https:// doi.org/10.1073/pnas.1817239116

2. D P Shivaprasad, Neetu Kumra Taneja, Anupama Lakra, Divya Sachdev. In vitro and in situ abrogation of biofilm formation in E. coli by vitamin C through ROS generation, disruption of quorum sensing and exopolysaccharide production. *Food Chem* 2021;**341**:128171. https://doi.org/10.1016/j.foodchem.2020.128171

3. Huicong Yan, Kyle L Asfahl, Na Li, Feng Sun, Junwei Xiao, Dongsheng Shen, et al. Conditional quorum-sensing induction of a cyanide-insensitive terminal oxidase stabilizes cooperating populations of *Pseudomonas aeruginosa*. *Nat Commun* 2019. https://doi.org/10.1038/s41467-019-13013-8

4. Tian Lu, Sobtop, Version 1.0, http://sobereva.com/soft/Sobtop.

5. López H, Puig S, Ganigué R, Ruscalleda M, Balaguer MD, Colprim J. Start-up and enrichment of a granular anammox SBR to treat high nitrogen load wastewaters. *J Chem Technol Biotechnol* 2008;**83**:233–241. https://doi.org/ 10.1002/jctb.1796

6. Li Y, Zheng P, Zhang M, Zeng Z, Wang Z, Ding A, et al. Hydrophilicity/hydrophobicity of anaerobic granular sludge surface and their causes: an in situ research. *Bioresour Technol* 2016;**220**:117–123. https://doi.org/ 10.1016/j.biortech.2016.08.012

7. Zhang S-H, Yu X, Guo F, Wu Z. Effect of interspecies quorum sensing on the formation of aerobic granular sludge. *Water Sci Technol* 2011;**64**:1284–1290. https://doi.org/10.2166/wst.2011.723

8. Miao L, Zhang Q, Wang S, Li B, Wang Z, Zhang S, et al. Characterization of EPS compositions and microbial community in an anammox SBBR system treating landfill leachate. *Bioresour Technol* 2018;**249**:108–116. https://doi.org/ 10.1016/j.biortech.2017.09.151

9. Bahram M, Bro R, Stedmon C, Afkhami A. Handling of rayleigh and raman scatter for PARAFAC modeling of fluorescence data using interpolation. *J Chemom* 2006;**20**:99–105. https://doi.org/10.1002/cem.978

10. Chen W, Westerhoff P, Leenheer JA, Booksh K. Fluorescence excitation−emission matrix regional integration to quantify spectra for dissolved organic matter. *Environ Sci Technol* 2003;**37**:5701–5710. https://doi.org/10.1021/ es034354c

11. Zhao Q, Li J, Deng L, Jia T, Zhao Y, Li X, et al. From hybrid process to pure biofilm anammox process: suspended sludge biomass management contributing to high-level anammox enrichment in biofilms. *Water Res* 2023;**236**:119959. https://doi.org/10.1016/j.watres.2023.119959

12. Smith CJ, Nedwell DB, Dong LF, Osborn AM. Diversity and abundance of nitrate reductase genes ( *narG* and *napA* ), nitrite reductase genes ( *nirS* and *nrfA* ), and their transcripts in estuarine sediments. *Appl Environ Microbiol* 2007;**73**:3612–3622. https://doi.org/10.1128/AEM.02894-06

13. Schmid M, Walsh K, Webb R, Rijpstra WI, van de Pas-Schoonen K, Verbruggen MJ, et al. *Candidatus* “scalindua brodae”, sp. nov., candidatus “scalindua wagneri”, sp. nov., two new species of anaerobic ammonium oxidizing bacteria. *Syst Appl Microbiol* 2003;**26**:529–538. https://doi.org/10.1078/ 072320203770865837

14. López-Gutiérrez JC, Henry S, Hallet S, Martin-Laurent F, Catroux G, Philippot L. Quantification of a novel group of nitrate-reducing bacteria in the environment by real-time PCR. *J Microbiol Methods* 2004;**57**:399–407. https://doi.org/ 10.1016/j.mimet.2004.02.009

15. Henry S, Bru D, Stres B, Hallet S, Philippot L. Quantitative detection of the nosZ gene, encoding nitrous oxide reductase, and comparison of the abundances of 16S rRNA, narG, nirK, and nosZ genes in soils. *Appl Environ Microbiol* 2006;**72**:5181–5189. https://doi.org/10.1128/AEM.00231-06

16. Tang X, Liu S, Zhang Z, Zhuang G. Identification of the release and effects of AHLs in anammox culture for bacteria communication. *Chem Eng J* 2015;**273**:184–191. https://doi.org/10.1016/j.cej.2015.03.045

17. Wang J, Ding L, Li K, Schmieder W, Geng J, Xu K, et al. Development of an extraction method and LC–MS analysis for N-acylated-l-homoserine lactones (AHLs) in wastewater treatment biofilms. *J Chromatogr B* 2017;**1041–1042**:37–44. https://doi.org/10.1016/j.jchromb.2016.11.029

1. ^*^Corresponding author

   *E-mail addresses*: xjniu@scut.edu.cn; scutenv@outlook.com (X. Niu), dqzhang3377@outlook.com (D. Zhang).

   *Address:* College of Environment and Energy, South China University of Technology, Guangzhou 510006, China [↑](#footnote-ref-2)
